# XAI-MRI: an ensemble dual-modality approach for 3D brain tumor segmentation using magnetic resonance imaging

**DOI:** 10.3389/frai.2025.1525240

**Published:** 2025-02-19

**Authors:** Ahmeed Suliman Farhan, Muhammad Khalid, Umar Manzoor

**Affiliations:** ^1^School of Computer Sciences, University of Hull, Hull, United Kingdom; ^2^Computer Center, University of Anbar, Ramadi, Iraq; ^3^School of Computer Science, University of Wolverhampton, Wolverhampton, United Kingdom

**Keywords:** ensemble dual-modality, brain tumor segmentation, MRI images, U-net, deep learning, convolutional neural network, Grad-CAM, XAI

## Abstract

Brain tumor segmentation from Magnetic Resonance Images (MRI) presents significant challenges due to the complex nature of brain tumor tissues. This complexity poses a significant challenge in distinguishing tumor tissues from healthy tissues, particularly when radiologists rely on manual segmentation. Reliable and accurate segmentation is crucial for effective tumor grading and treatment planning. In this paper, we proposed a novel ensemble dual-modality approach for 3D brain tumor segmentation using MRI. Initially, individual U-Net models are trained and evaluated on single MRI modalities (T1, T2, T1ce, and FLAIR) to establish each modality's performance. Subsequently, we trained U-net models using combinations of the best-performing modalities to exploit the complementary information and improve segmentation accuracy. Finally, we introduced the ensemble dual-modality by combining the two best-performing pre-trained dual-modalities models to enhance segmentation performance. Experimental results show that the proposed model enhanced the segmentation result and achieved a Dice Coefficient of 97.73% and a Mean IoU of 60.08%. The results illustrate that the ensemble dual-modality approach outperforms single-modality and dual-modality models. Grad-CAM visualizations are implemented, generating heat maps that highlight tumor regions and provide useful information to clinicians about how the model made the decision, increasing their confidence in using deep learning-based systems. Our code publicly available at: https://github.com/Ahmeed-Suliman-Farhan/Ensemble-Dual-Modality-Approach.

## Introduction

Brain tumors represent one of the most severe and complex challenges in the medical field (Muhammad et al., 2021). They arise from abnormal growth of cells within the brain or inside the skull (Farhan et al., 2023). It is one of the most dangerous diseases and one of the leading causes of death in various countries (Alqhtani et al., 2024). The most common type of brain tumors is gliomas, which are considered a challenge for both doctors and researchers because of diversity and difficulty in diagnosing and treating them. It is estimated that about 80,000 people in the United States are diagnosed with brain tumors each year, and the majority of them have gliomas (Zhou, 2023; Thakkar et al., 2020). Gliomas are classified into two main groups according to their grade: high-grade gliomas (HGG) and low-grade gliomas (LGG). Determining the grade has a significant role in planning the treatment (Sun et al., 2023). Although low-grade gliomas are less aggressive than high-grade gliomas, they can develop into higher-grade gliomas if there is no treatment in time, which increases the severity of the disease and complicates treatment (Bogdańska et al., 2017; Claus et al., 2015). Therefore, early diagnosis of brain tumors is essential and increases the chances of patients survival and treatment (Al-Zoghby et al., 2023; Saeedi et al., 2023). Diagnosis of brain tumors begins with MRI because it is the most efficient tool for imaging brain tissue. In addition, MRI provides a three-dimensional view of the brain, which helps doctors better diagnose the tumor (Abd-Ellah et al., 2024).

**Figure 1:**
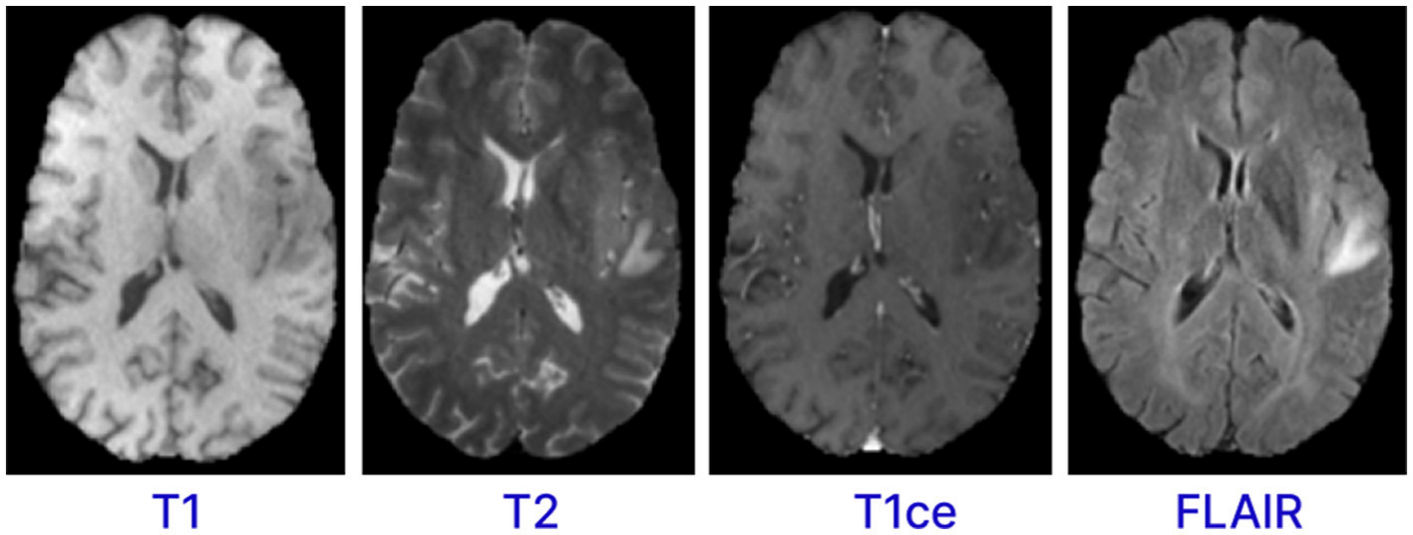
MRI sequences for brain.

Magnetic resonance imaging (MRI) is a form of multimodality imaging. MRI generates images of different contrasts because protons in the tissues vary in their relaxation rates (Zhan et al., 2022; Zhou et al., 2019). Different modalities of MRI images, such as T1-weighted, T1-weighted images with contrast enhancement (T1c), FLAIR, and T2-weighted help doctors better view the tumor (Tandel et al., 2023). For example, T2 and FLAIR modalities target on the edema area surrounding the tumor while T1 and T1c focus on the tumor area (Zhou et al., 2019). Figure 1 shows the different modalities of brain MRI images. These different modalities help doctors accurately analyze and diagnose the tumor and develop treatment plans (Hammad et al., 2023; Sailunaz et al., 2023). However, brain tumors from MRI images can be difficult to recognize and segment due to high variability in tumor shape, size, and location (Almufareh et al., 2024). In addition, manual segmentation is time-consuming and prone to errors (due to variability in interpretations) and requires an expert radiologist. Therefore, there is an urgent need to develop an automatic system for segmenting brain tumors from MRI images. This system can help segment and diagnose tumors accurately and efficiently (Karim et al., 2024; Hussain and Shouno, 2024).

Accurately segmenting brain tumors from magnetic resonance imaging (MRI) data is important to diagnosis. However, segmentation is complicated due to the different brain tumors types and the difficulty distinguishing between infected and healthy cells, highlighting the urgent need for an automated segmentation system. In recent years, deep learning, especially the U-Net architecture, has shown promising results in medical image segmentation (Zhang F. et al., 2024). Because each modality provides limited information, single-modality MRI methods often do not perform well in brain tumor segmentation. Although multiple MRI modalities can enhance segmentation accuracy, most current segmentation models do not integrate features from different modalities. This study suggests a new ensemble dual-modality method based on the U-Net model. This method aims to improve segmentation performance by training and integrating bimodal U-Net models, making the method for brain tumor segmentation more accurate. The study shows that the proposed ensemble dual-modality model performed better than the single-modality and multi-modality models. This study aims to address the following research questions:

- How can the ensemble dual-modality model improve the robustness of segmentation models?
- How can the model enhance explainability and build clinical trust?
- How can interactive tools and feedback mechanisms facilitate real-world adaptation and continuous learning of segmentation models across different clinics and enhance the generalization of the segmentation model?

The main contributions of this study is as follows:

- An ensemble dual-modality module is proposed to combine two pre-trained dual-modality models to improve segmentation accuracy.
- This study evaluates the results of the U-net model on single-modality input and multi-modalities input.
- Explainable AI techniques such as Grad-CAM is used to enhance interpretability and thus enable clinicians to understand decision-making.
- An interactive interface with feedback mechanisms is built to facilitate the feedback mechanism, enabling the model to continuously learn from data from different clinics, enhancing the model's generalizability.
- The study proposed a novel approach for preprocessing 3D MRI images and cropping the region of interest (ROI). Accurately identifying and cropping the region of interest reduces the complexity of segmentation and ensures that the segmentation model focuses on the most relevant areas of the brain.
- The proposed method was rigorously evaluated using the BraTS2020 dataset and the results shows the effectiveness of the ensemble dual-modality model in accurately segmenting brain tumors.

## Literature review

In recent years, the field of medical image analysis has gained significant attention, particularly in the segmentation of brain tumor from MRI images. The deep learning models has become one of the most famous methods in medical image segmentation because of its strong ability to extract features. A lot of research has been done in the field of using the deep learning models for brain tumor segmentation, and has shown its efficiency and usefulness in tumor segmentation.

A significant trend in brain tumor segmentation involves using multiple MRI modalities to enhance segmentation results for example, Zhou (2024) proposed a novel multi-modalities brain tumor segmentation based on U-net, which utilized disentangled representation learning and region-aware contrastive learning. The disentangled representation learning helps to disentangle the entangled features into independent factors that represent various components of the tumor, and the region-aware contrastive learning helps to mine the feature representation from the related tumor regions. The proposed model was evaluated on the BraTS 2018 and BraTS 2019 datasets and achieved outperforming compared with other state-of-the-art models. Similarly, Zhou (2023) proposed a new brain tumor segmentation multi-modalities model from MRI images. The model has the ability to segment brain tumors even when one or more modalities are missing and can also reconstruct the missing modalities. It exploits multiple MRI modality features in order to enhance segmentation performance. Furthermore, latent feature learning is employed to extract multimodal latent correlations. The proposed approach was evaluated on the BraTS 2018 dataset and achieved promising segmentation results. In a comparable manner, Zhu et al. (2024) proposed an approach that uses multi-modality MRI data to enhance spatial information and obtain more accurate tumor boundary. This approach includes three modules: (1) border shape correction (BSC), (2) spatial information enhancement (SIE), and (3) modality information extraction (MIE). Together, these modules are assembled into 3D brain tumor segmentation model that works on the input, backbone, and loss functions of DCNNs. The proposed model evaluated on BraTS2017, BraTS2018, and BraTS2019 datasets. The proposed model achieved an average DSC of 0.821, 0.858, and 0.853 for the BraTS2017, BraTS2018, and BraTS2019 datasets, respectively. As well, Ranjbarzadeh et al. (2024) proposed a brain tumor segmentation framework using four modalities of MRI image types. The proposed model is based on convolutional neural networks (CNNs) and Improved Chimp Optimization Algorithm (IChOA). Initially, all four MRI modality types (T1, T2, T1ce, and Flair) are normalized to identify potential tumor regions. Afterwards, IChOA is used to select features using a Support Vector Machine (SVM) classifier. Finally, these features are fed into the proposed CNN model for tumor segmentation. IChOA contributes to feature selection and hyperparameter optimization of the proposed CNN model. Experimental results on the BRATS 2018 dataset show that the proposed model achieved a Precision of 97.41%, recall of 95.78%, and dice score of 97.04%.

Some researchers have focused on improving computational efficiency while maintaining reasonable segmentation accuracy. Montaha et al. (2023) introuced a 2D U-Net-based approach to segmenting brain tumors from 3D MRI data. It utilizes skip connections to preserve spatial information and processes 2D slices from the 3D scans to reduce computational costs while maintaining high performance. The preprocessing steps, such as rescaling and normalization, further enhance performance. The proposed method is trained and tested on the BraTS2020 dataset, and the model achieved 93.1% DSC and 99.41% accuracy respectively. As well, Feng et al. (2024) introduced a new way to represent frequencies to reduce feature loss in the segmentation model, primarily when brain tumor detection is encoded and decoded. This method, called MLU-Net, integrates frequency representation techniques and Multilayer Perceptron (MLP)-based techniques into a lightweight U-Net architecture. MLU-Net enhances the performance of medical image segmentation tasks while maintaining high computational efficiency by using frequency representation and MLP-based methods. Experimental results for brain tumor segmentation highlight the significant advantages of the proposed approach. MLU-Net achieved remarkable efficiency improvements, reducing parameters and computational workload to just 1/39 and 1/61 of those required by the U-Net model. Also, it outperforms U-Net in segmentation accuracy, improving the Dice and Intersection over Union (IoU) metrics by 3.37% and 3.30%, respectively. Also, Zhang W. et al. (2024) proposed a novel approach named ETUNet (Efficient Transformer Enhanced UNet) for 3D brain tumor segmentation. This method integrates transformer modules into the UNet architecture to utilize their efficiency in capturing long-range dependencies and enhancing feature representations. By incorporating transformers, ETUNet aims to improve the segmentation accuracy and efficiency compared to UNet models. The proposed approach evaluated on BraTS-2018 and BraTS-2020 datasets, and obtained average Dice Similarity Coefficient (DSC) scores of 0.854 and 0.862 and Hausdorff Distance (HD95) values of 6.688 and 5.455 on BraTS-2018 and BraTS-2020 datasets, respectively.

To address the challenge of limited labeled data (Hammer Håversen et al., 2024) proposed a novel approach for 3D segmentation using a self-supervised and self-querying framework integrated into the U-Net architecture. Unlike traditional methods that rely solely on labeled data for supervision, QT-UNet utilize self-supervision to learn from unlabelled data, reducing the need for annotated samples. The proposed approach evaluated on (BraTS 2021) dataset and obtained a Hausdorff Distance of 4.85 mm and an average Dice score of 88.61.

Other studies involve the introduction of dual-path models. For example, Fang and Wang (2022) proposed a dual-path network designed to enhance the effectiveness of brain tumor segmentation. This architecture used data from multiple MRI modalities to improve segmentation accuracy in MRI images. The proposed model is trained and evaluated on the BraTS 2015 dataset, demonstrating efficient performance.

To enhance the model explainability (Dasanayaka et al., 2022b) proposed an interpretable machine learning model based on U-Net and DenseNet for brain tumor segmentation and classification from MRI images. The segmentation model segments the brain tumors into enhancing tumor, whole tumor, and tumor cor. The segmentation model achieved Dice coefficients of 0.779, 0.885, and 0.804 for enhancing tumor, whole tumor, and tumor core, respectively. The classification module classifies the tumor into three types: glioblastoma, oligodendroglioma, and astrocytoma and achever an average accuracy of 89.3% and a kappa coefficient of 0.733. They used Grad-CAM to explain how the model makes decisions, enhancing its transparency and reliability for medical use.

To Improving feature fusion and contextual learning, Yousef et al. (2023) proposed a novel Deep Learning-based Brain Tumor Segmentation architecture called Bridged-U-Net-ASPP-EVO for Brain tumor segmentation and includes spatial hierarchical pooling (ASPP) and an advanced normalization layer. The modifications to the on U-Net architecture incorporates include: (1) an Atrous Spatial Pyramid Pooling (ASPP) module, which captures multi-scale information, and a bridging mechanism that facilitates better feature fusion between the encoder and decoder paths. (2) The Evolving Normalization Activation Layer (EVO_NORM) is utilized to optimize feature normalization and activation simultaneously, leading to better convergence and accuracy. (3) The model integrates a Squeeze and Excitation with Residual Block (SE-Block) to recalibrate channel-wise feature responses and strengthen relevant features while reducing noise. (4) A Bridge Layer is introduced to improve information flow between the encoder and decoder paths, ensuring precise spatial and contextual feature fusion. The proposed approach is tested and evaluated using the BraTS 2020 and BraTS 2021 datasets. The evaluation results on the BraTS 2020 dataset showed an average of 0.78, 0.8159, and 0.9073 for ET, TC, and WT, respectively, and HD95% of 21.684, 15.941, and 5.37. The test results on the BraTS 2021 dataset achieved averaged DSC of 0.8434, 0.8594, and 0.9187 for ET, TC, and WT, respectively, and average HD95% of 11.669, 141887, and 5.3687.

While the studies mentioned have showcased the effectiveness of U-net for brain tumor segmentation, however, the existing approaches often face limitations in leveraging the complementary information from multiple MRI modalities with a multi-path model. Additionally, existing approaches may suffer from robustness and reduced generalization capability. These studies highlight the following challenges:

- Lack of robustness and generalization: Models generally suffer from reduced generalizability when applied to different datasets, which limits their clinical usefulness. This issue becomes even more apparent when data comes from different devices or clinics. Since models trained on a single dataset often fail to generalize well to differences in imaging protocols.
- Limited explainability: Existing models often lack mechanisms for explaining their predictions, which is very important in medical applications.
- Real-world implementation: Many current approaches lack tools, interactive interfaces, or frameworks that allow clinicians to interact with model outputs or allow feedback or use of real-world data in the training process. This prevents the model from being flexible enough to generalize across different clinics and devices. Deep learning models can only succeed in the real world if they learn from large real-time datasets from multiple clinics and imaging devices.

To address these limitations, our proposed ensemble Dual-Modality approach integrates different MRI modalities and ensemble Dual paths to enhance segmentation accuracy. By leveraging the strengths of each modality and combining them through an ensemble framework, we aim to improve the robustness and efficiency of brain tumor segmentation while mitigating the challenges associated with single-modality approaches. Moreover, using Grad-CAM gives clear insights into how the model makes the decisions and ensures that predictions are explainable, bridging a gap in explainable AI for medical applications.

Additionally, we designed an interactive user interface that facilitates the model's real-world application. This interface enables clinicians to upload MRI data, review segmentation results, and provide annotations or corrections. The feedback collected through this interface is incorporated into the training process, allowing the model to adapt to real-world data from multiple clinics and devices. By creating a continuous learning loop, the tool ensures the model generalizes effectively across diverse clinical environments, addressing both robustness and real-world implementation challenges.

## Proposed methodology

**Figure 2:**
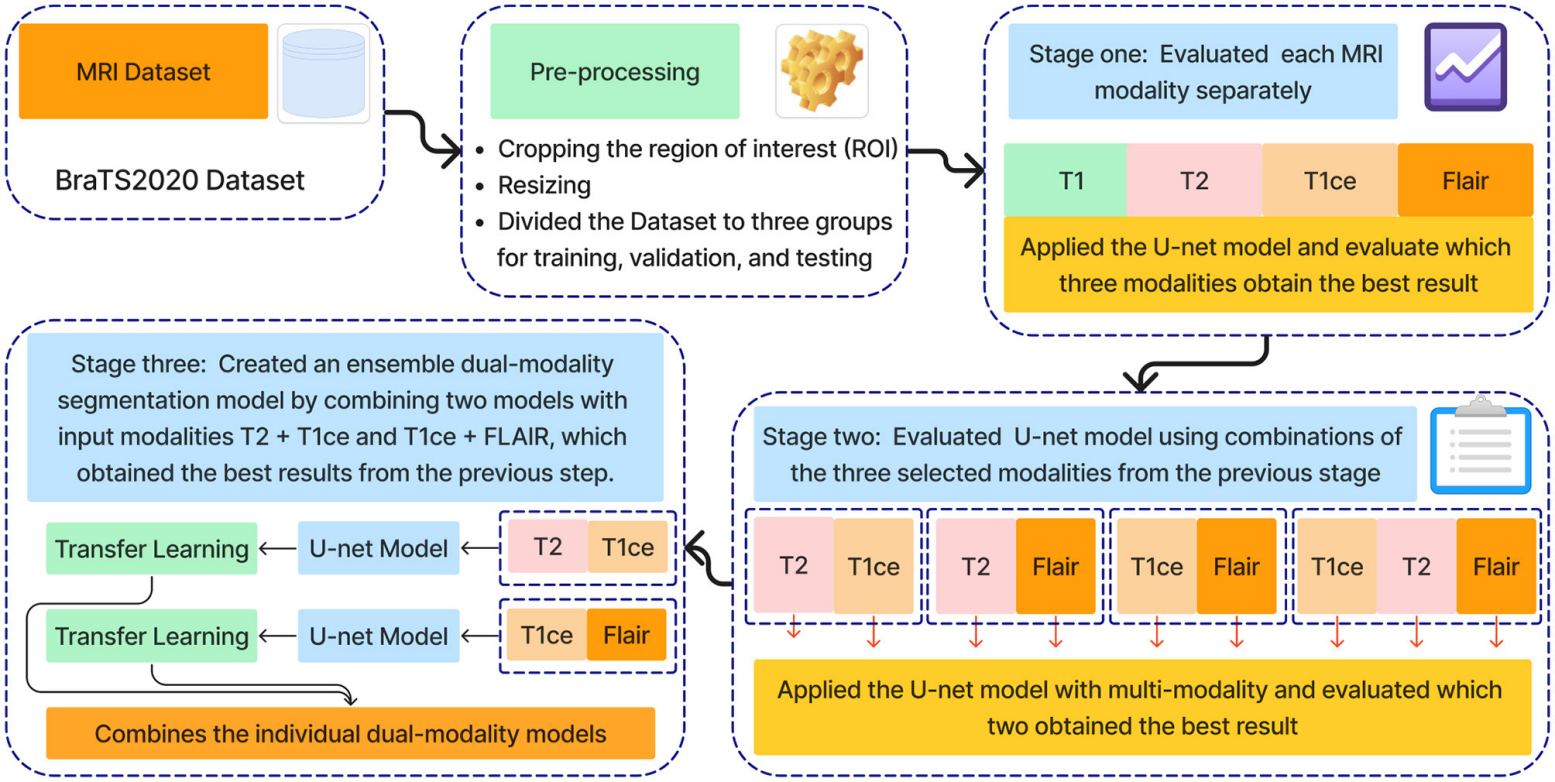
Workflow of the proposed approach.

The methodology proposed in this research is an ensemble dual-modality approach for brain tumor segmenting from MRI images. Figure 2 shows all stages of the proposed technology. In the first stage, the BraTS2020 dataset was chosen to train and test the proposed method. The second stage of pre-processing the dataset includes identifying and cropping the region of interest and resizing it, then dividing it into testing, training, and validation sets. After that, the third stage is feature extraction and segmentation of the brain tumor, comprising three key steps: Step one trains and evaluates the U-net model on each MRI modality separately, including T1, T2, T1ce, and FLAIR. The second step involves training and evaluating the U-net model using multi-modalities. The third step combined two pre-train models with input modalities T2+T1ce and T1ce+FLAIR, which obtained the best results from the previous step in creating an ensemble dual-modality segmentation model. Together, these stages form a comprehensive framework for enhancing the efficiency of brain tumor segmentation. The following sections explain each stage in more detail, including its impact on brain segmentation performance.

### U-Net model architecture

**Figure 3:**
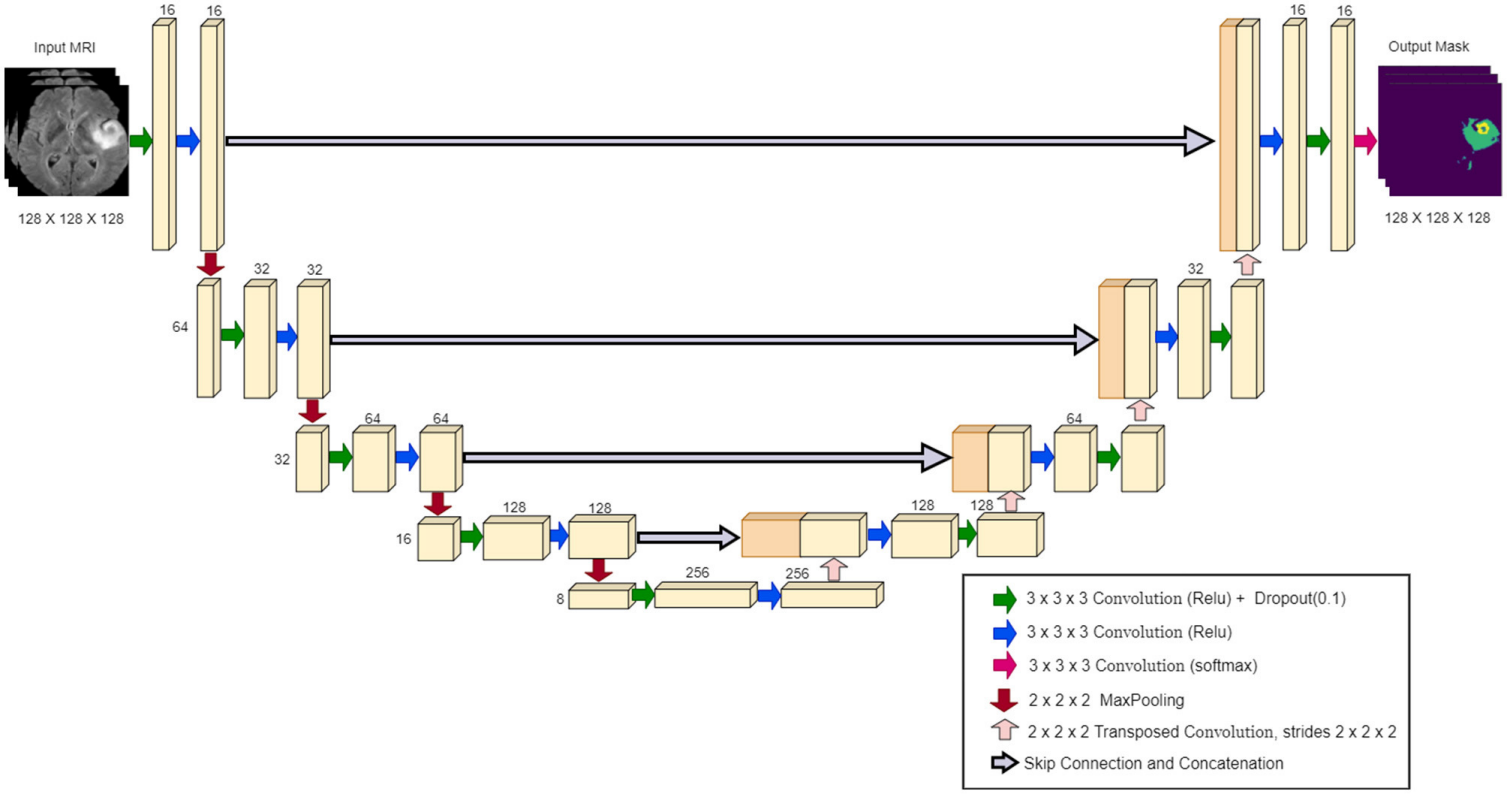
U-Net model architecture.

The U-Net architecture is a convolutional neural network widely used in biomedical image segmentation. It was introduced by Ronneberger et al. (2015), and is characterized by a symmetrical “U” shape, consisting of a contracting path (encoder) and a dilating path (decoder). The encoder captures the context through convolutional layers and max-pooling layers, gradually reducing the spatial dimensions as depth increases. The decoder then upsamples the features, using the transferred convolutions, and connects them to the corresponding encoder features through skip connections (Ibtehaz and Rahman, 2020; Montaha et al., 2023). Figure 3 illustrates the specific configuration of the 3D U-Net architecture utilized in our study, highlighting its layers and connections.

### Datasets

This study used the Brain Tumor Segmentation 2020 (BraTS2020) dataset to train and test the proposed approach. This dataset contains 494 3D image subjects, divided into 369 subjects for training and 125 for validation. Each subject includes MRI scan data across multiple modalities: T1-weighted, T1 post-contrast (T1ce), T2-weighted, and FLAIR (fluid-attenuated inversion recovery). Each modality has a resolution of 240 × 240 in the axial plane, paired with a depth of 155 slices. The training dataset also contains ground truth labels that have been manually reviewed by doctors. Therefore, in this study, we used these 369 subjects for training and testing the proposed method. The annotations ground truth include [tumor core (TC), whole tumor (WT), and enhanced tumor (ET)] (Menze et al., 2014; Bakas et al., 2017, 2018).

### Pre-processing

The preprocessing of the BraTS2020 dataset includes cropping the region of interest (ROI). The ROI cropping step is important and improves the performance of segmentation models by making the model focus on the relevant parts of the brain. This study used 369 subjects from the BraTS2020 dataset to train and test the model. These 369 subjects include the ground truth labels.

**Figure 4:**
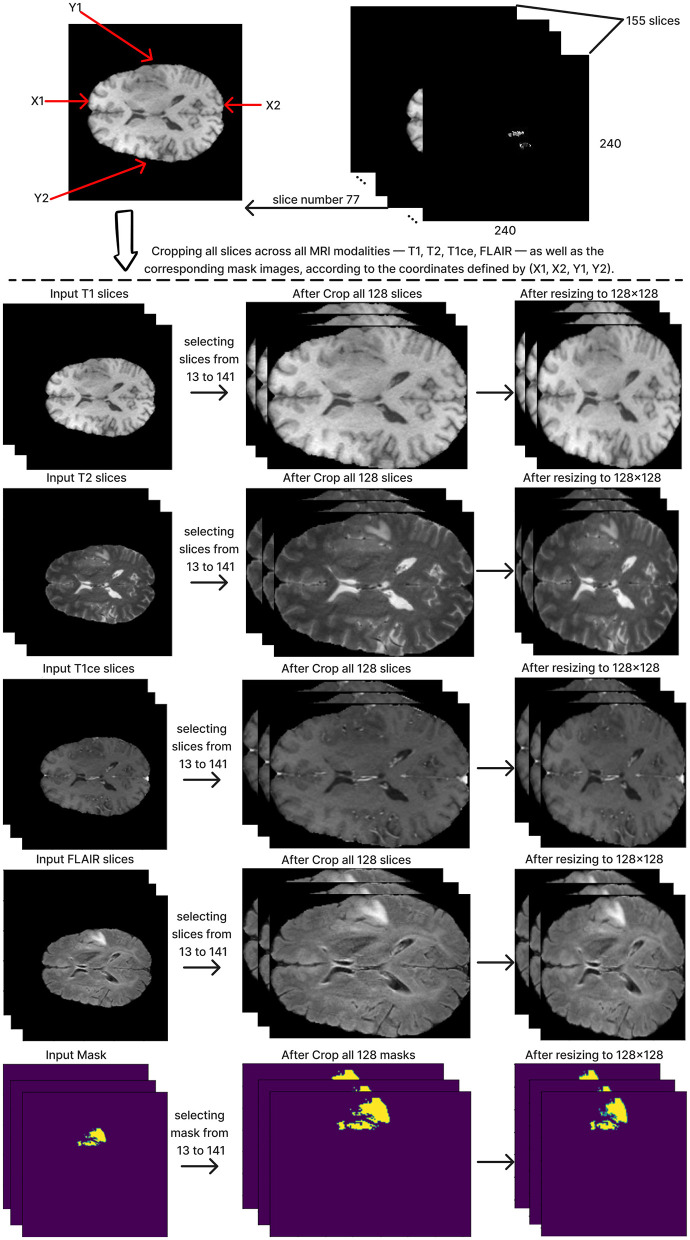
Description preprocessing steps.

To define and crop the ROI for each subject, we chose the T1 modality of MRI images because this modality demonstrates the contrast between the brain and the surrounding edges. Slice number 77 was chosen from the T1 modality, which includes a depth of 155 slices. As the middle slice, this typically offers a central view of the brain and the tumor, ensuring that the cropped region in all other slices encompasses the whole brain. This slice was used to calculate the coordinates x1, x2, y1, and y2, which are critical to determining the ROI. Subsequently, we reduced the depth from 155 to 128 slices by selecting slices from 13 to 141. The next step is cropping all slices by applying the same calculated coordinates value on all 128 slices of the MRI across all modalities: T1, T2, T1ce, FLAIR, and the mask. After that, the cropped images were resized to 128x128 pixels, resulting in a standardized input size of (128, 128, 128) for the segmentation model. Figure 4 shows these steps in detail.

Finally, the dataset was divided into three groups for training, validation, and testing. The distribution was 70% of the dataset for training, 15% for validation, and 15% for testing.

### Ensemble dual-modality method

**Figure 5:**
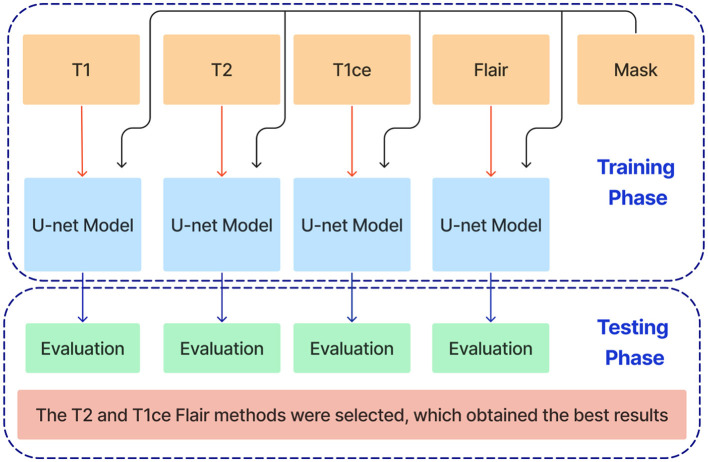
Training and evaluation of individual U-net models on separate MRI modalities (T1, T2, T1ce, and FLAIR) for brain tumor segmentation.

This paper introduces an ensemble dual-modality approach to enhance brain tumor segmentation capabilities and help clinical decision-making. Individual Unet models are trained and evaluated on each MRI modality separately, including T1, T2, T1ce, and FLAIR. This step determines each method's essential tumor segmentation performance, as shown in Figure 5. The T2 and T1ce Flair methods were selected, which obtained the best results for the next step.

**Figure 6:**
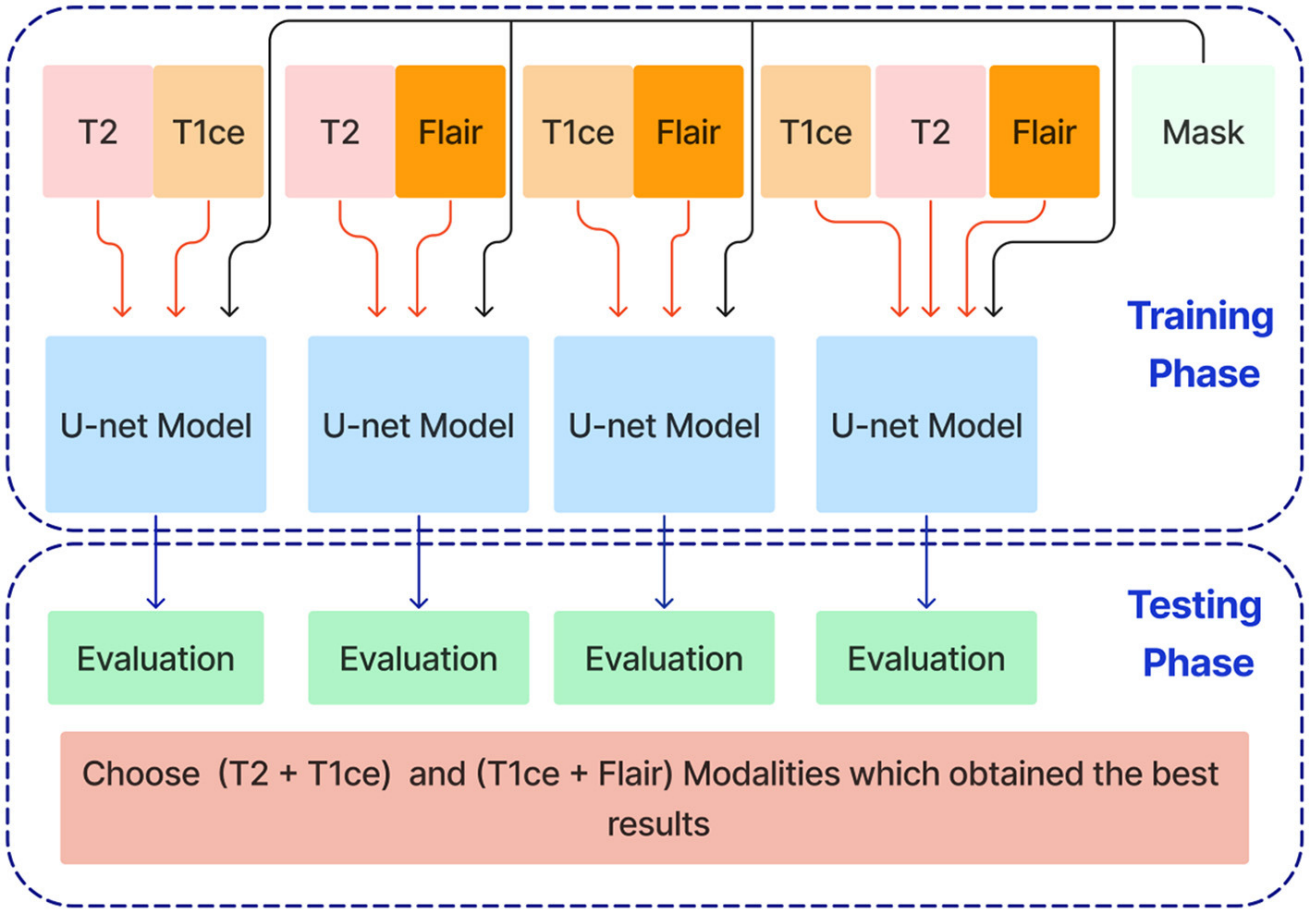
Training and evaluation U-net models using combinations of selected MRI modalities (T2 + T1ce, T2 + FLAIR, T1ce + FLAIR, T2 + T1ce + FLAIR) for enhanced brain tumor segmentation.

In the next stage, Unet models are trained using combinations of the three selected modalities, which obtained the best results from the previous step. This method trains models on multi-input modalities, such as T2 + T1ce, T2 + FLAIR, T1ce + FLAIR, T2 + T1ce + Flair, as shown in Figure 6. This method exploits complementary information in different modalities to enhance segmentation accuracy. Using multiple modalities simultaneously can yield more robust segmentation results because each MRI modality captures unique aspects of tumor characteristics.

**Figure 7:**
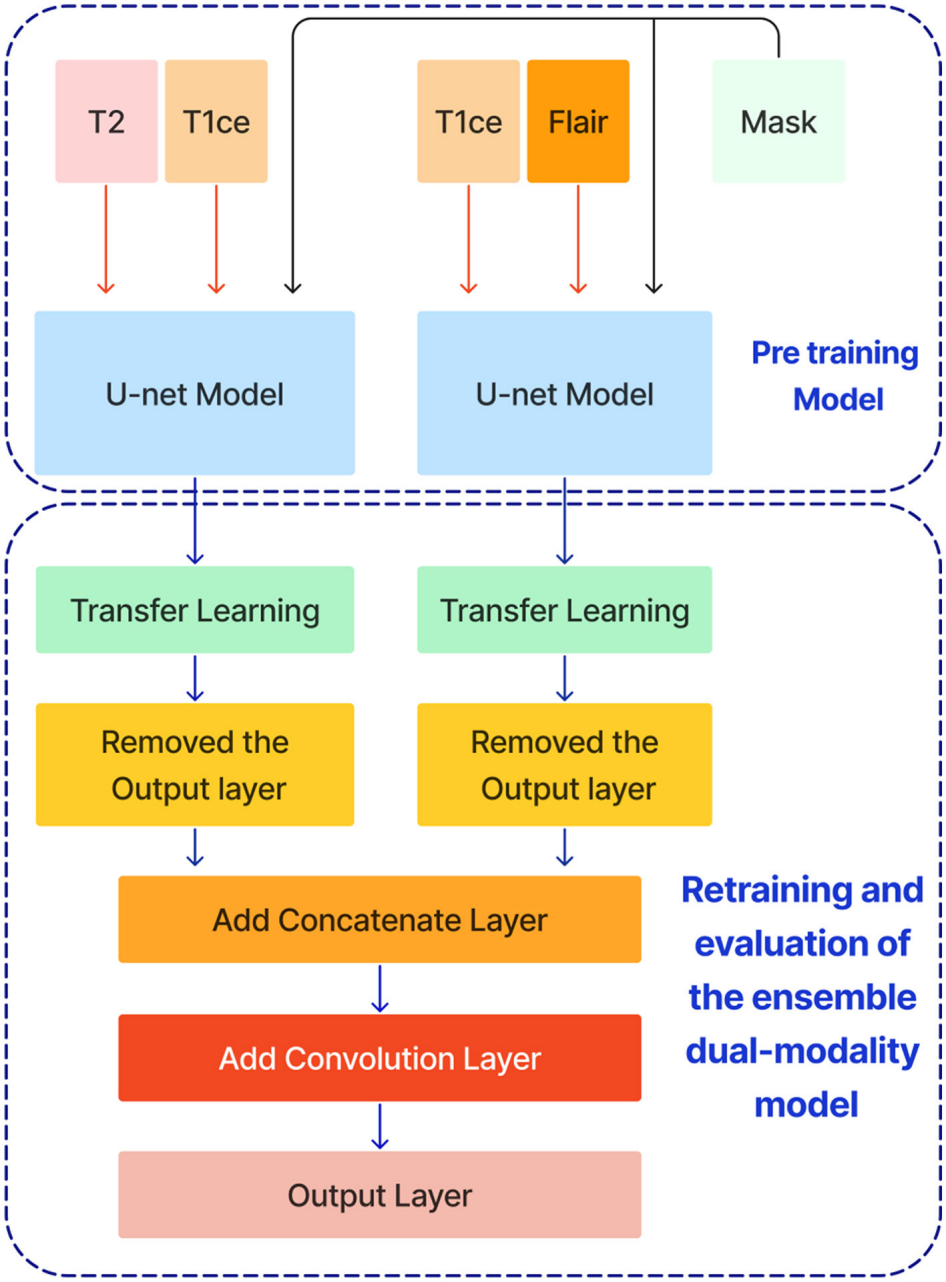
The ensemble dual-modality segmentation model by combining the best-performing dual-modality models (T2 + T1ce and T1ce + FLAIR).

After training the dual modality models, the pre-trained models are combined. We chose the two models with input modalities T2 + T1ce and T1ce + FLAIR that obtained the best results from the previous steps to create an ensemble dual-modality segmentation model. This ensemble process combines the individual dual-modality models by removing the output layer from each model and adding a Concatenate Layer to concatenate their feature maps. They were followed by an additional convolutional layer with [a Relu activation function, a filter size of (3 × 3 × 3), a stride size of 1 and an output shape of 16] Afterwards, an output layer with a softmax activation function as shown in Figure 7. The Ensemble Dual-Modality method aims to harness the complementary strengths of each modality, leading to a more comprehensive and accurate segmentation of brain tumors. The Ensemble Dual-Modality approach integrates multiple MRI modalities and ensemble the Unet deep learning model to improve the accuracy and reliability of brain tumor segmentation.

### Visualizations and interactive interface

We have developed an interactive web-based interface to facilitate specialist and clinic access. The tool provides functions such as managing brain MRI data, reviewing system predictions, predicting tumor diagnostics, and offering an interactive interface for specialist feedback on tumor predictions, all of which require user registration. The tool features a main dashboard that displays key statistics, including the total number of MRI images uploaded, those processed by the system, those yet to be reviewed by specialists, and those already reviewed. Users can upload brain MRI images through the data upload interface, with unique identification numbers generated for each patient to ensure privacy and tracking. The search and review interface allows users to view processed MRI predictions and see details of segmented tumor areas and annotations. An interactive interface lets specialists modify and perform manual tumor segmentation and provide detailed notes. These inputs can retrain the system with real-world data from various clinics, enhancing its accuracy and generalisability over time.

## Experiment results

This section comprehensively overviews the specific hyperparameters used in the experiments. It also describes the performance metrics used to demonstrate the effectiveness of the ensemble dual-modality model in segmenting brain tumors from MRI images. It then details the ensemble dual-modality testing results on the BraTS 2020 dataset.

### Experimental setup

The proposed model was implemented using Python 3.8.0, Keras 2.6.0, and TensorFlow 2.6.0 libraries. The experiments were performed on a device with the following specifications: OS [Linux: Ubuntu 22.04.4 LTS (GNU/Linux 5.15.0-97generic x86_64)], Processor [Intel(R) Xeon(R) CPU E52680 v4 @ 2.40GHz], RAM (128 GB) and GPU [05:00.0 VGA compatible controller: Matrox Electronics Systems Ltd. MGA G200e [Pilot] ServerEngines (SEP1) (rev 05)].

### Hyperparameters

Before starting the training process, basic hyperparameters must be set, including batch size, number of epochs, and learning rate. For this task, the batch size was 4, the number of epochs was 40, and the learning rate was 0.0001. There was also an early stopping point for stopping training if validation loss did not improve after five consecutive epochs. This strategy helps to avoid overfitting and improves computational efficiency by aborting training when further development is not likely. These weights with the smallest validation loss were saved and used to test the approach.

### Performance metrics

To evaluate the performance of our brain tumor segmentation models, we used several key metrics: accuracy, mean intersection over union (Mean IoU), Dice similarity coefficient (DSC), precision, sensitivity, and specificity. These measures show you how the proposed approach performs from different aspects (Verma et al., 2024; Jyothi and Singh, 2023; Renugadevi et al., 2023).

1. **Accuracy:** The segmentation accuracy is based on the proportion of correctly classified pixels (tumor and non-tumor) in comparison with total pixels. Accuracy, although helpful for generalizing performance, it can be misleading in imbalanced data, such as segmenting brain tumors, where pixels from non-tumor often trump tumor pixels.
  (1) $$\begin{array}{l} Accuracy=\frac{\left(TP+TN\right)}{\left(TP+TN+FP+FN\right)} \end{array}$$
2. **Mean intersection over union (Mean IoU):** The mean IoU is a very important metric for evaluating the accuracy of the segmentation. It measures the mean overlap between the predicted and ground truth mask. The equation gives the mean IoU:
  (2) $$\begin{array}{l} IoU=\frac{\left|A⋂B\right|}{\left|A⋃B\right|} \end{array}$$
3. **Dice similarity coefficient:** The DSC is another important metric that measures the similarity between the predicted segmentation and the ground truth. It is defined as twice the overlap area divided by the total number of pixels in the predicted and ground truth masks. The equation gives DSC:
  (3) $$\begin{array}{l} Dice=2\frac{\left|A⋂B\right|}{\left|A\right|+\left|B\right|} \end{array}$$
  where *A* is the set of pixels in the predicted segmentation, and *B* is the set of pixels in the ground truth.
4. **Precision:** Precision measures the accuracy of the model's positive predictions, defined as the ratio of actual positive pixels to the sum of true positive and false positive pixels. It indicates the number of predicted tumor pixels that belong to the tumor.
  (4) $$\begin{array}{l} Precision=\frac{TP}{\left(TP+FP\right)} \end{array}$$
5. **Sensitivity (Recall):** Sensitivity or recall measures a model's ability to correctly identify all true positive pixels. It is the ratio of true positive pixels to the sum of true positive and false negative pixels.
  (5) $$\begin{array}{l} Sensitivity=\frac{TP}{\left(TP+FN\right)} \end{array}$$
6. **Specificity:** Specificity measures a model's ability to correctly identify all true negative pixels. The ratio of true negative pixels to the sum of true negative and false positive pixels.
  (6) $$\begin{array}{l} Specificity=\frac{TN}{\left(TN+FP\right)} \end{array}$$

While all of these metrics are important, we primarily focused on the mean IoU and Dice Similarity Coefficient (DSC) in our study. These metrics are more useful for evaluating segmentation tasks because they directly measure the overlap between predicted and actual tumor regions, providing a clearer picture of the model's performance in accurately defining tumor boundaries.

### Results

**Figure 8:**
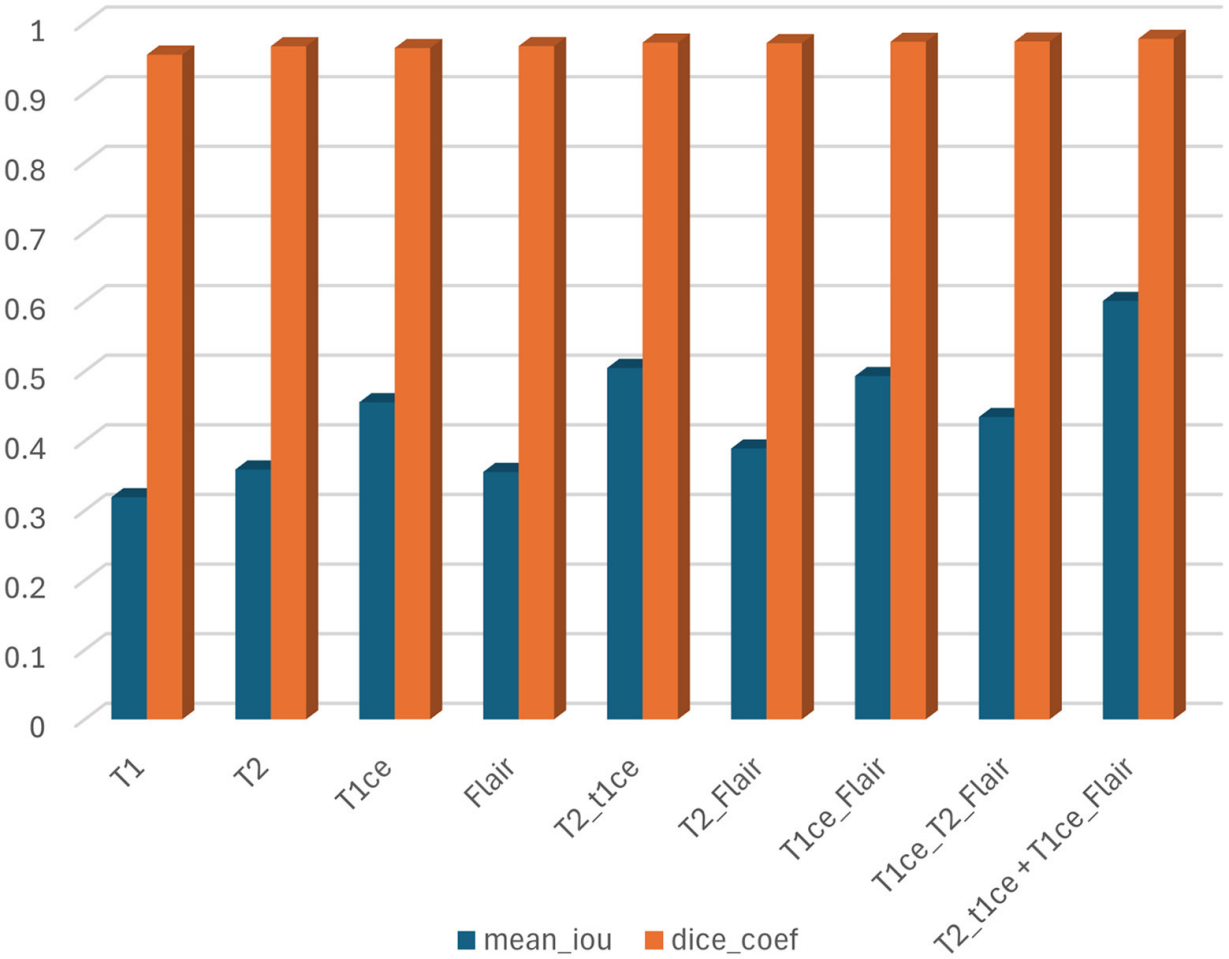
Results of mean IoU and DSC for all scenarios.

In this section, we trained and tested the proposed model using the BraTS2020 dataset, which includes T1, T1c, T2, and FLAIR modalities. This experiment was divided into three scenarios: single modality, multi-modality, and ensemble dual-modality. Table 1 and Figure 8 show the summary of the test results for all scenarios.

**Table 1:** Results for all scenarios.

**Metrics**	**T1**	**T2**	**T1ce**	**Flair**	**T2_t1ce**	**T2_flair**	**T1ce_flair**	**T1ce_T2 _Flair**	**T2_t1ce + T1ce_flair**
Accuracy	0.969191	0.975229	0.974564	0.975452	0.979207	0.978127	0.981185	0.979357	**0.983575**
Mean_iou	0.318932	0.358804	0.455298	0.355311	0.504634	0.388971	0.493028	0.434116	**0.600833**
Dice_coef	0.954508	0.966633	0.964015	0.966846	0.971875	0.971087	0.972989	0.973409	**0.977302**
Precision	0.984345	0.986528	0.981337	0.981470	0.983169	0.984929	0.987366	0.983540	**0.987980**
Sensitivity	0.957838	0.965744	0.969547	0.971912	0.976294	0.973914	0.976221	0.976706	**0.980297**
Specificity	0.994924	0.995619	0.993877	0.993903	0.994438	0.995052	0.995851	0.994563	**0.996028**

Bold values indicate the best-performing results for each metric.

#### Single-modality segmentation

In this scenario, each MRI modality (T1, T2, T1ce, and FLAIR) was trained and tested on the U-Net model. The brain tumor segmentation performance was diverse among different MRI modalities during training and testing. The T1 modality achieved a Dice Coef of 0.954508 and a Mean IoU of 0.318932. Meanwhile, the T2 modality showed a Dice Coef of 0.966633 and a Mean IoU of 0.358804. In contrast, the T1ce modality obtained a Dice Coef of 0.964015 and a Mean IoU of 0.455298; T1ce images enhanced the visibility of the tumor due to contrast enhancement. Finally, the FLAIR modality resulted in a Dice Coef of 0.966846 and a Mean IoU of 0.355311. T1ce demonstrated the highest Mean IoU, indicating its potential for better tumor delineation. In addition, the T2 and FLAIR modalities performed better than the T1 modality. These results indicate that while single modalities provide valuable information, they have limitations in accurately capturing the full extent of the tumor.

#### Multi-modality segmentation

U-Net models were trained using combinations of multiple MRI modalities. Combining modalities improved segmentation accuracy significantly, as shown in Table 1 and Figure 8. The T2 with T1ce modality achieved a Dice Coef of 0.971875 and a Mean IoU of 0.504634. While T2 with FLAIR, this combination resulted in a Dice Coef of 0.971087 and a Mean IoU of 0.388971. In contrast, the T1ce with FLAIR Has a Dice Coef of 0.972989 and a Mean IoU of 0.493028. Conversely, the T2 + T1ce + FLAIR triple-modality achieved a Dice Coef of 0.976191 and a Mean IoU of 0.579291. This multi-modality approach leveraged the complementary information provided by different MRI sequences, leading to more comprehensive tumor segmentation.

#### Ensemble dual-modality segmentation

**Figure 9:**
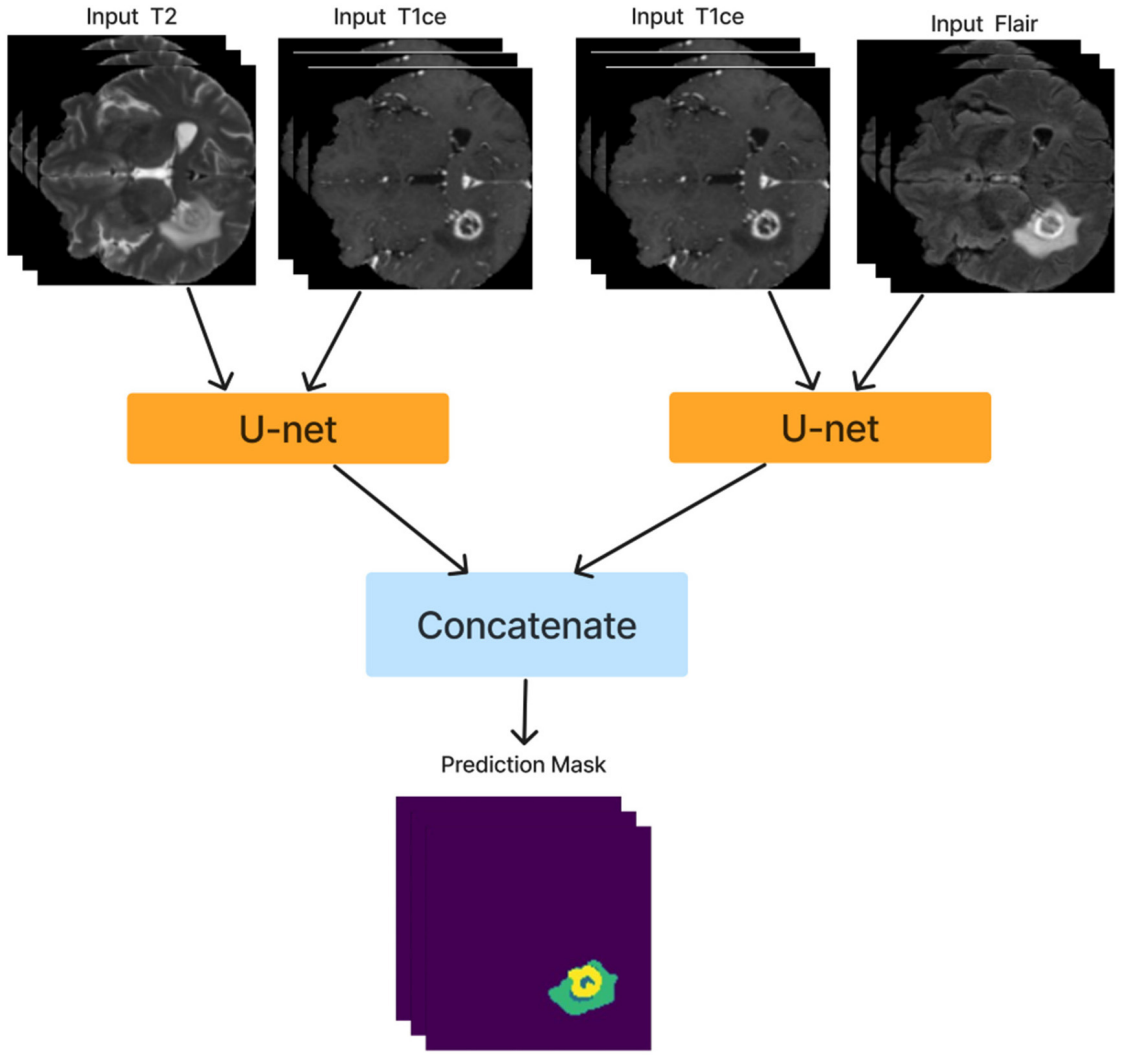
Example of applying the ensemble dual-modality method.

The Ensemble Dual-Modality model combined the pre-trained models of the two best-performing dual-modality combinations (T1ce+FLAIR and T2+FLAIR). The Ensemble Dual-Modality model outperformed both the single-modality and multi-modality models by achieving a Dice Coef of 0.977302 and a Mean IoU of 0.600833. The Ensemble Dual-Modality model provided more accurate tumor segmentation because it integrated features from both dual-modality models, leveraging the strengths of each model. Also, using additional convolutional layers in the Ensemble Dual-Modality model helped merge features from different models, thus improving performance. Figure 9 shows an example of the result of the Ensemble Dual-Modality model applied.

**Figure 10:**
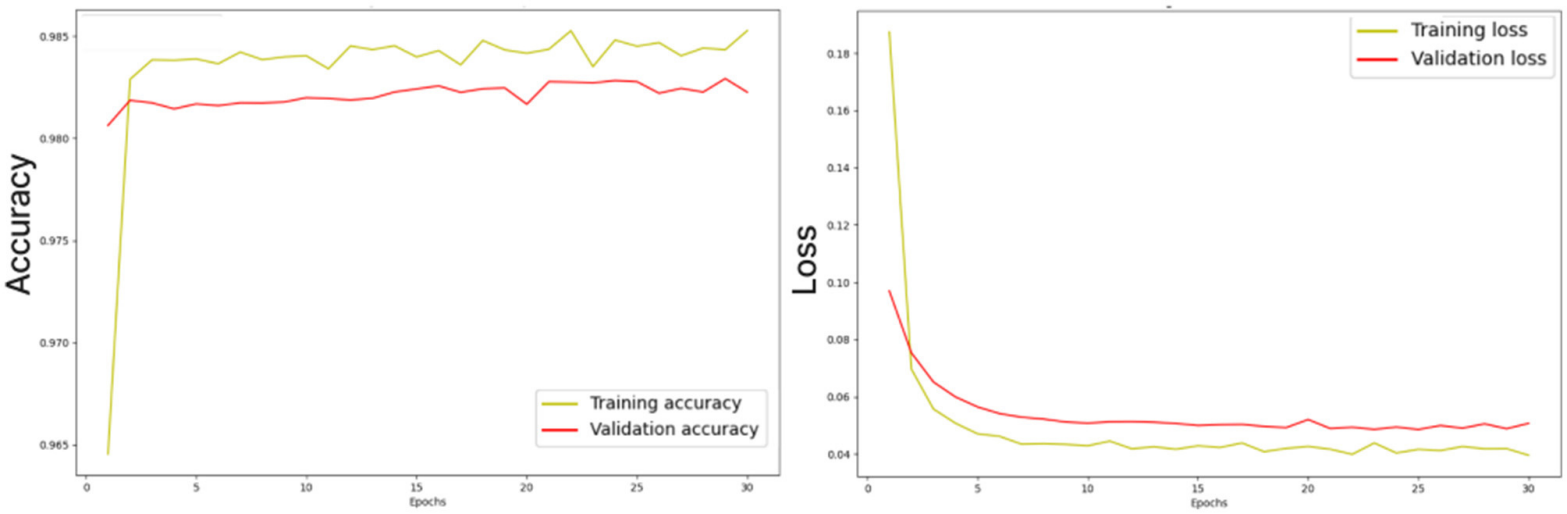
Losses and accuracy curves of the ensemble dual-modality model during the training and validation process.

Figure 10 illustrates the losses and accuracy curves of the ensemble dual-modality model during the training and validation process. The left plot shows the trends of accuracy, where it can be seen that the training accuracy increases smoothly and stabilizes above 98%, while the validation accuracy follows this trend, indicating that overfitting is minimal. The right plot shows the training and validation loss curves. The training loss drops rapidly in the first few epochs and then converges to a stable value. While the validation loss follows a trend close to the training loss, indicating that the model can generalize to unseen data.

### Compare the proposed ensemble dual-modality approach with existing methods

In this comparative study, we compare the result of the proposed ensemble dual-modality with the following standard models: U-Net (Ronneberger et al., 2015), U-Net++ (Zhou et al., 2018), and Attention U-Net (Oktay et al., 2018). For a fair evaluation, the same dataset (BraTS2020) was used, and all hyperparameters and server settings were the same. The results in Table 2 show the proposed ensemble dual-modality approach outperforms the other models. Our model achieved a Dice Coefficient of 0.977 and a Mean IoU of 0.601, While U-Net obtained (Dice: 0.973, Mean IoU: 0.434), U-Net++ (Dice: 0.969, Mean IoU: 0.445), and Attention U-Net (Dice: 0.971, Mean IoU: 0.424). The integration of complementary features from the dual-modality models and extra convolutional layer led to increased segmentation accuracy.

**Table 2:** Comparison of the proposed ensemble dual-modality model with existing other models.

**Methodology**	**Dice coefficient**	**Mean IoU**
U-Net (Ronneberger et al., 2015)	0.973	0.434
U-Net++ (Zhou et al., 2018)	0.969	0.445
Attention U-Net (Oktay et al., 2018)	0.971	0.424
Ensemble dual-modality (Proposed)	0.977	0.601

### Explainability

The black box behavior of AI algorithms has been questioned, with the quest to see how predictions are made. Especially in medicine, doctors are skeptical about blindly accepting predictions without a proper understanding (Wijethilake et al., 2021). Therefore, explainability is very important when applying AI models in clinical applications. It makes the decision-making process of the AI model understandable and trustworthy for clinicians. In this study, we embedded explainability using Grad-CAM visualizations (Selvaraju et al., 2017; Farhan et al., 2023; Dasanayaka et al., 2022a) and web interactive user interfaces.

**Figure 11:**
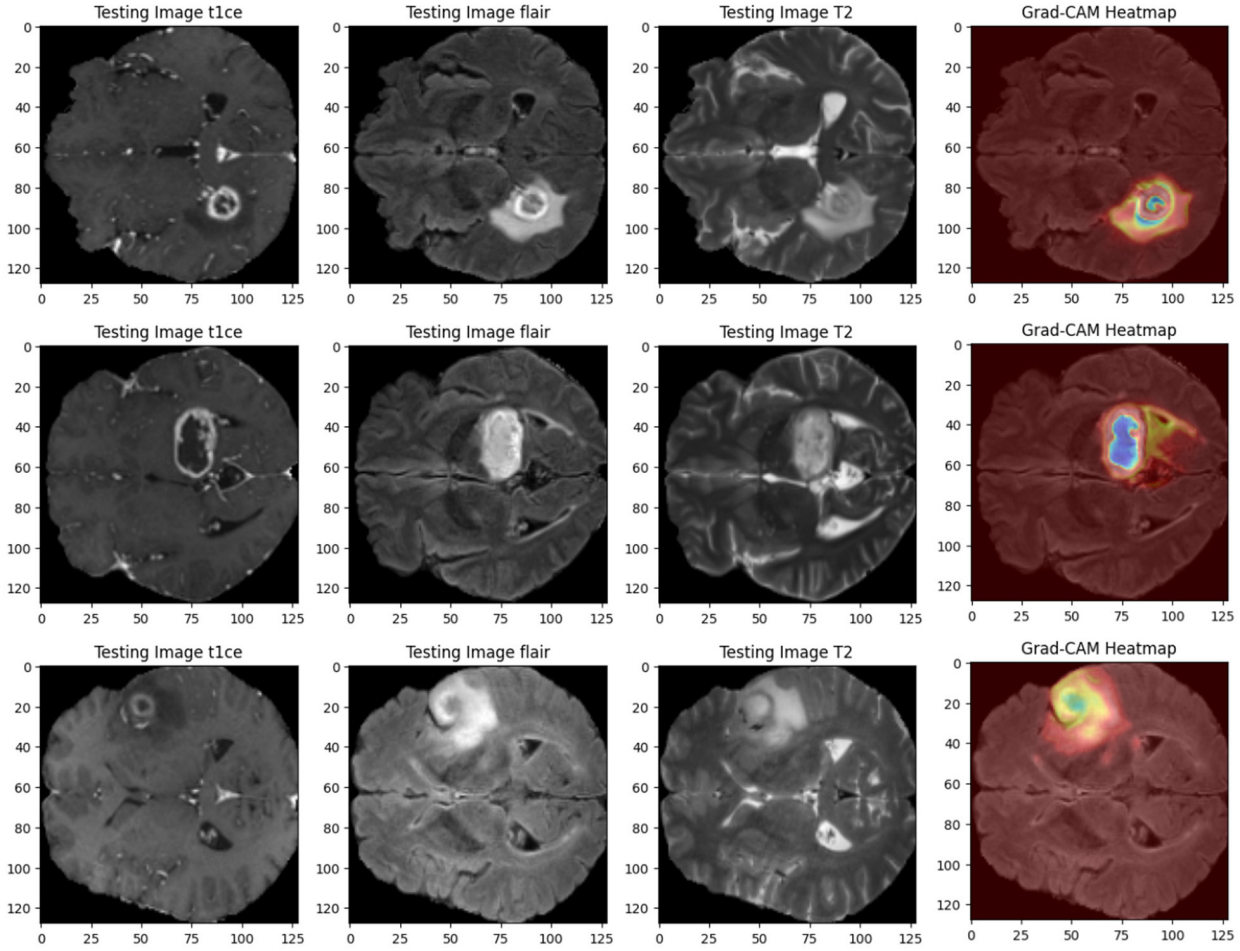
Visualization of MRI input images alongside Grad-CAM heat maps.

**Figure 12:**
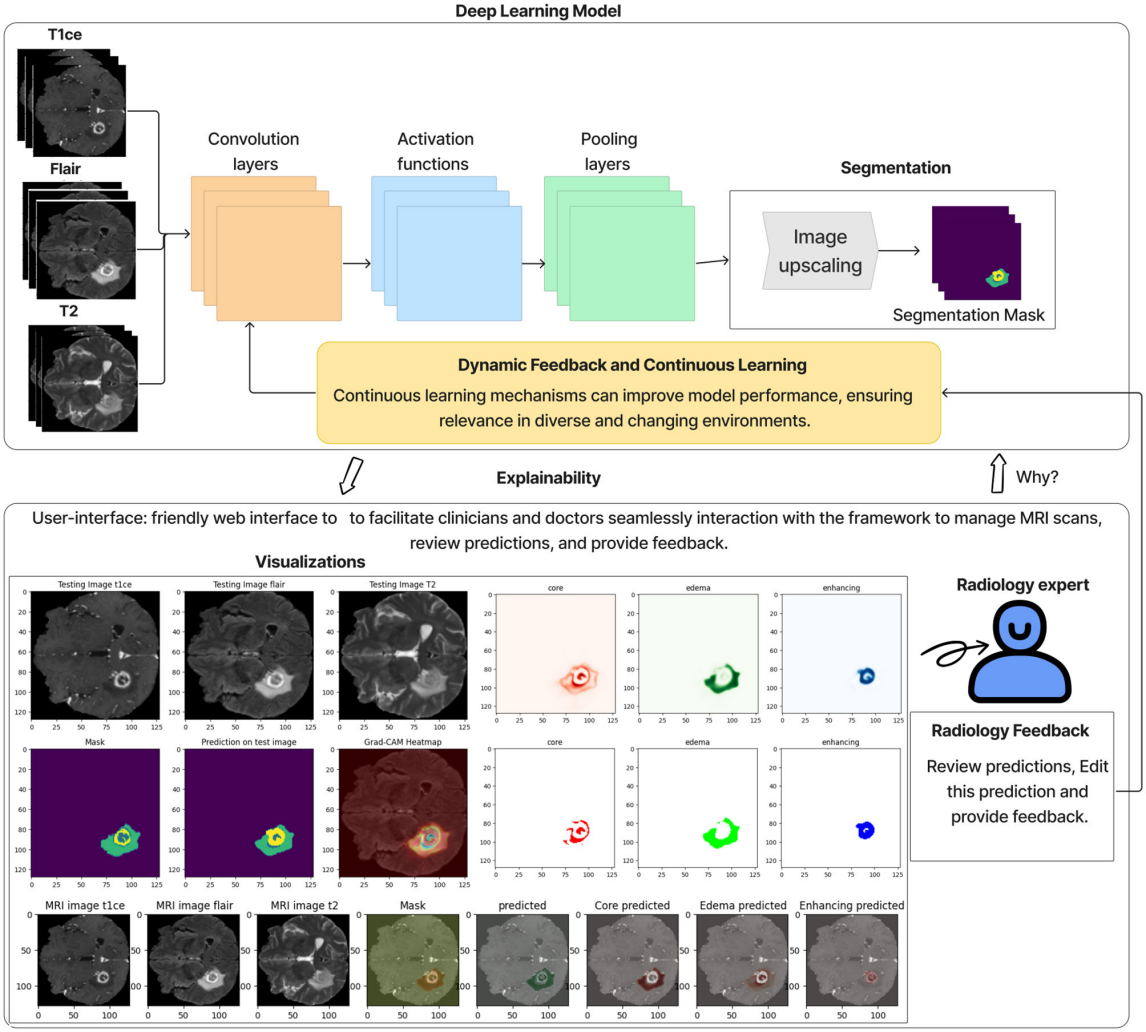
An interactive visualization tool for brain tumor segmentation.

- Grad-CAM heat maps: Grad-CAM heat maps pinpoint the regions within the MRI scans that the model focuses on when segmenting. These visualizations overlay the heat maps on top of the original images, giving clear insights into how the model makes its decisions. Figure 11 shows an example of Grad-CAM visualizations for the dual-modality ensemble model, where regions of the tumor are highlighted. This approach enhances explainability and helps doctors verify the model's predictions.
- Interactive visualization tool and feedback: We developed an interactive user interface on the web to display the brain tumor segmentation results. This tool provides clinicians with a user-friendly interface to:
  1- Review the segmentation results.
  2- Annotate and modify segmentations interactively.
  3- Provide feedback on predictions, such as correcting segmentation boundaries or adding comments.
  The feedback is used to retrain the model, improving its accuracy and generalizability over time. Figure 12 illustrates an example of this interactive interface.

This explainability ensures that the proposed model is accurate, transparent, and adaptable to clinical workflows, fostering trust and confidence among healthcare professionals.

## Discussion

These results show that the proposed ensemble dual-modality model is effective in improving accuracy and robustness in brain tumor segmentation. The proposed model achieved a Dice coefficient of 0.977 and an average IoU of 0.601 respectively, by leveraging complementary information from multiple MRI modalities, outperforming standard methods such as U-Net and U-Net++. Moreover, the visualizations shown in Supplementary Figures 1–9 present the full segmentation outcomes for the different modalities and provide further evidence of the robustness of our segmentation approach.

In order to improve the explainability of the proposed method, Grad-CAM visualizations are implemented, generating heat maps highlighting tumor regions. This functionality gives useful information to clinicians on the model's decision processes, increasing their trust in using deep learning-based systems. An interactive user interface is also developed to get feedback on decisions such as modify the segmentation results obtained by the model or annotations, or correcting the predictions. This feedback contributes to the continuous retraining of the model on data from various clinics and different imaging protocols, which leads to model generalizability. This feature makes the proposed model more applicable to real-world applications and addresses the limitations of many existing approaches, such as robustness and generalizability.

The performance of the proposed model is compared with U-Net, U-Net++, and Attention U-Net, under identical conditions (same database, hyperparameters, and hardware specifications). As shown in Table 2, the ensemble dual-modality model achieved superior segmentation results over the these models, and this improvement is attributed to the ability of the ensemble framework to effectively combine the strengths of multiple models.

The trade-off between model complexity and performance, the ensemble dual-modality framework is carefully designed to balance resource usage with improved segmentation accuracy. By only including the best two performing dual-modality models, T1ce + FLAIR and T2 + T1ce, we optimized the integration of complementary information while keeping computational complexity manageable. Furthermore, parallel processing during training and ROI cropping reduced the memory and computational overhead.

The study also considered potential challenges such as overfitting and underfitting during the training process. To mitigate overfitting, we implemented dropout layers and used early stopping to prevent overtraining. Underfitting is addressed by designing the ensemble dual-modality approach to incorporate complementary information from dual-modality inputs, ensuring that the model captures a broader range of tumor features. Monitoring the learning curves (training accuracy and validation/loss) confirmed the signs of overfitting or underfitting. These curves, as shown in Figure 10, show a close alignment between the training and validation metrics, indicating a well-trained model.

While the study relied on the BraTS2020 dataset (for training, validation, and testing), future work will rely on collaboration with physicians to evaluate and apply the model in the real world. The proposed approach can be tested on real-world data and its effectiveness in clinical practice. The feedback mechanism by experts will play an important role in retraining the model and thus continuous improvement over time.

## Conclusion

Reliable and accurate segmentation is crucial for effective tumor grading and subsequent treatment planning. Different MRI sequences such as T1, FLAIR, T1ce, and T2 provide unique insights into various aspects of the tumor. Our study proposed a novel ensemble dual-modality approach for 3D brain tumor segmentation from MRI. The proposed approach leverages the strengths of multiple MRI modalities and ensemble learning. The results demonstrate that the U-net model with single-modality input can be significantly enhanced through dual-modality and ensemble methods. By combining T2, T1ce, and FLAIR modalities, dual-modality achieved better performance than single-modality in terms of Dice Coefficient and Mean IoU, underscoring the value of utilizing complementary information from different imaging techniques. The ensemble dual-modality model combined the two dual-modality pre-trained models that achieved the best results. The proposed approach achieved a Dice coefficient of 97.73% and a Mean IoU of 60.08% when evaluated on the BraTS2020 dataset. The proposed method leverages the strengths and characteristics of each modality to obtain accurate segmentation. The results indicate the potential of leveraging ensemble learning in other medical applications that involve complex diagnoses.

## Data Availability

Publicly available datasets were analyzed in this study. This data can be found here: https://www.kaggle.com/datasets/awsaf49/brats20-dataset-training-validation.

## Supplementary material

Supplementary Figure 1 Example of segmentation results with just T1 modality input.

Supplementary Figure 2 Example of segmentation results with just T2 modality input.

Supplementary Figure 3 Example of segmentation results with just T1ce modality input.

Supplementary Figure 4 Example of segmentation results with just flair modality input.

Supplementary Figure 5 Example of segmentation results with T2 + T1ce modalities input.

Supplementary Figure 6 Example of segmentation results with T2 + flair modalities input.

Supplementary Figure 7 Example of segmentation results with T1ce+ flair modalities input.

Supplementary Figure 8 Example of segmentation results with T2 + T1ce + flair modalities input.

Supplementary Figure 9 The segmentation results example of ensemble dual-modality approach.
