# Supplementary figures and images for: XAI-MRI: an ensemble dual-modality approach for 3D brain tumor segmentation using magnetic resonance imaging
Source: Front Artif Intell. 2025 Feb 19;8:1525240. doi: 10.3389/frai.2025.1525240 (PMC11880613; doi:10.3389/frai.2025.1525240)

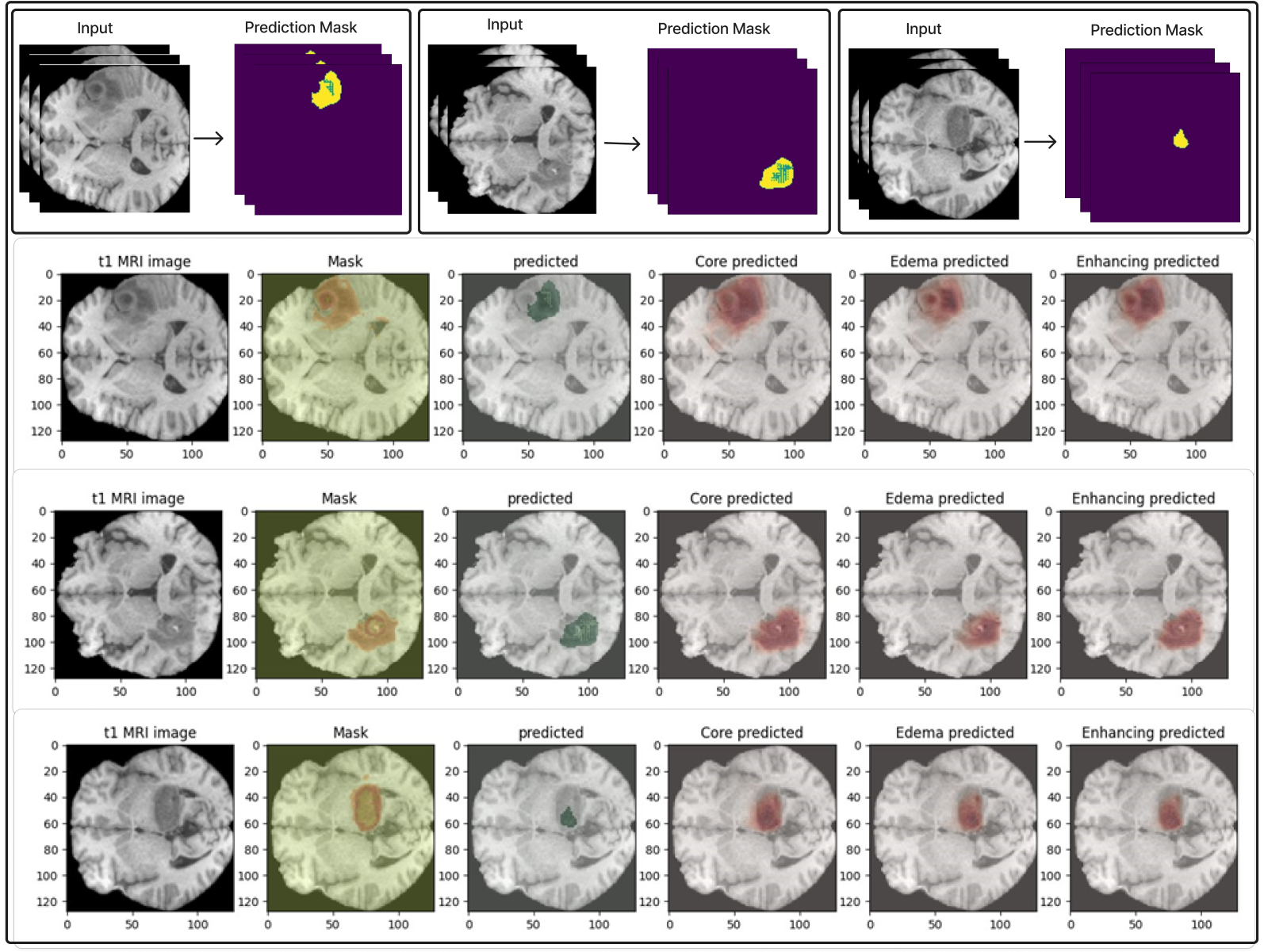

Supplement: Supplementary Figure 1 — Example of segmentation results with just T1 modality input. [file Data_Sheet_1.zip › Supplementary Material/Figure 1.png]

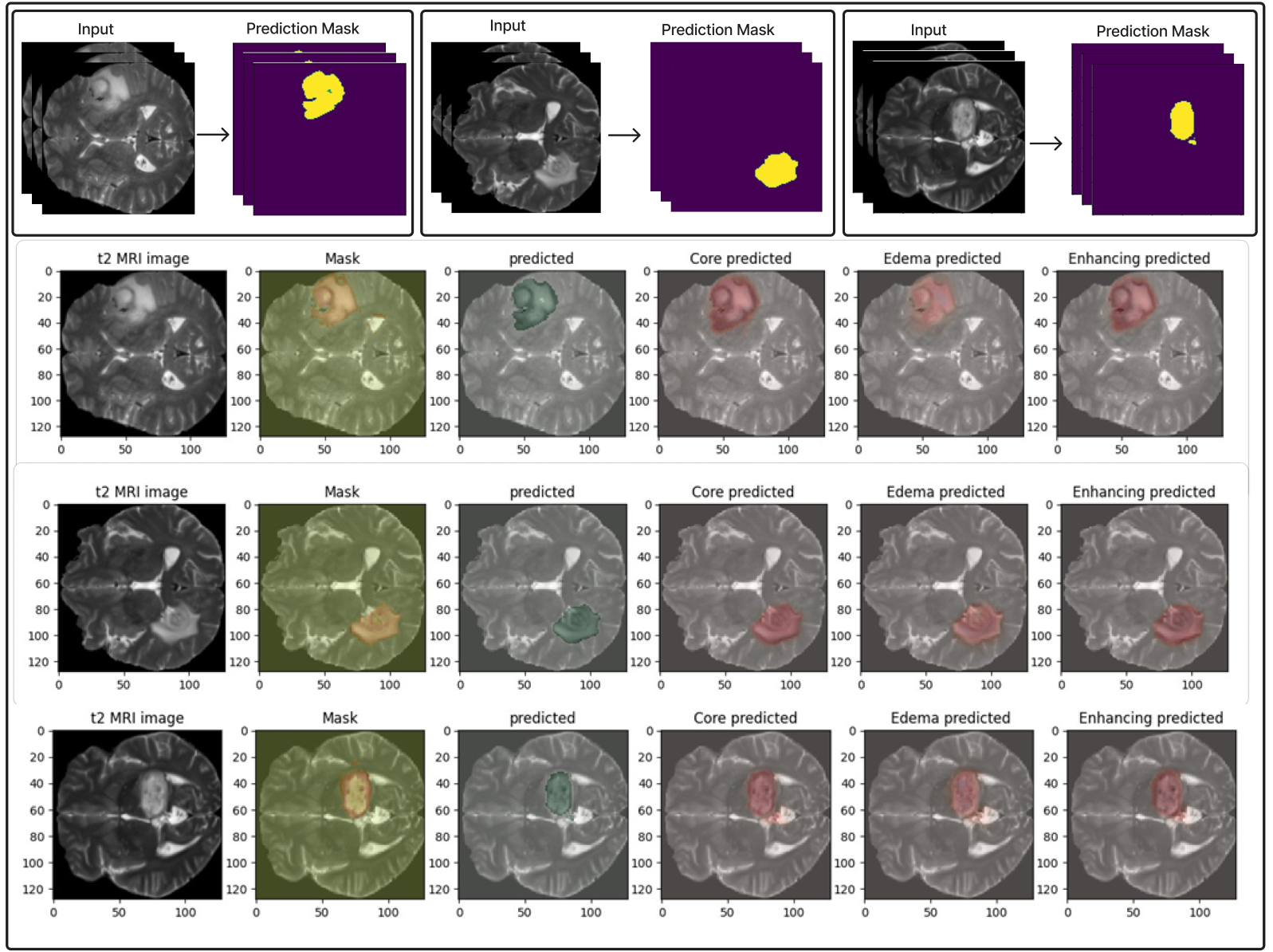

Supplement: Supplementary Figure 1 — Example of segmentation results with just T1 modality input. [file Data_Sheet_1.zip › Supplementary Material/Figure 2.png]

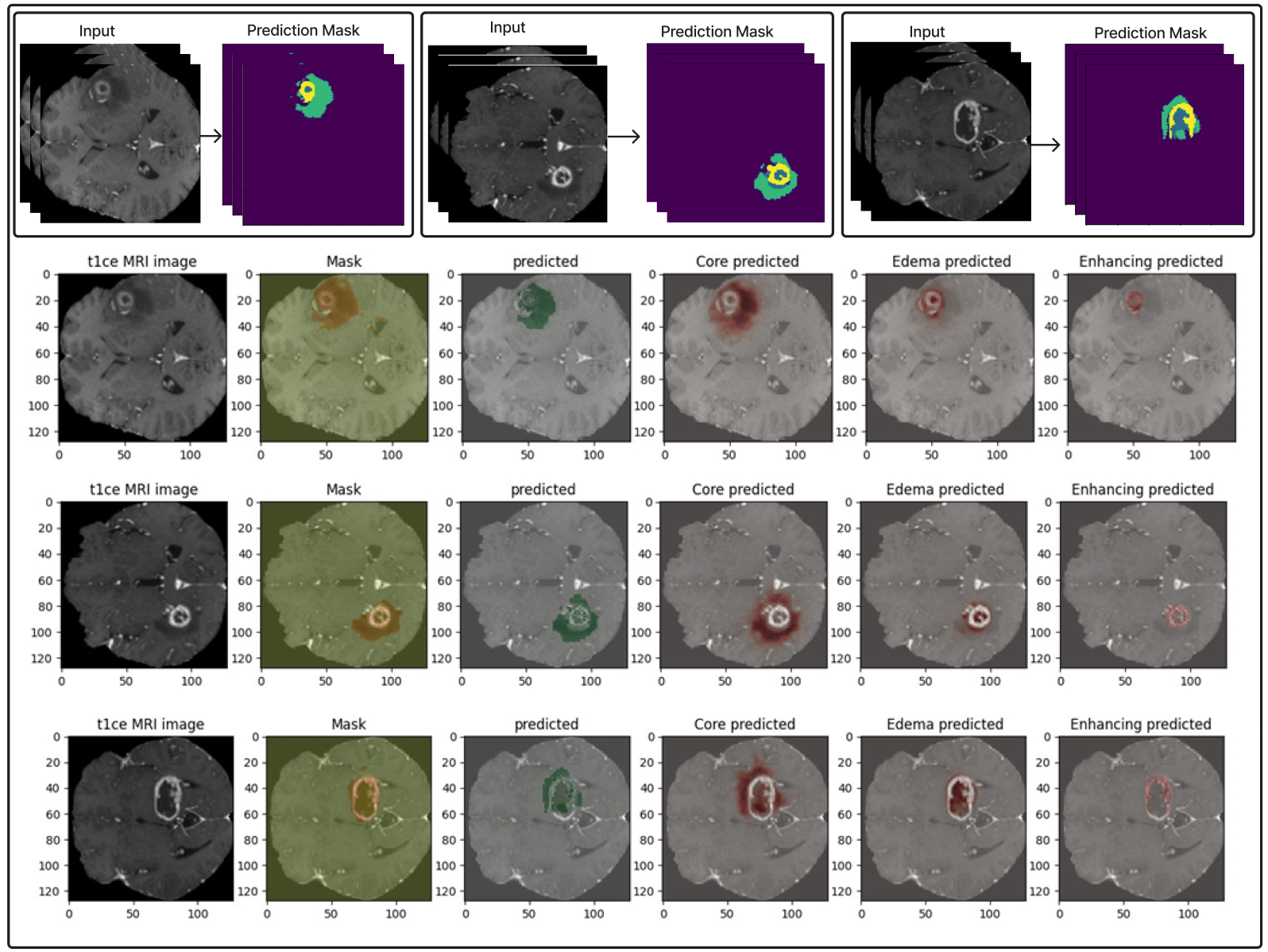

Supplement: Supplementary Figure 1 — Example of segmentation results with just T1 modality input. [file Data_Sheet_1.zip › Supplementary Material/Figure 3.png]

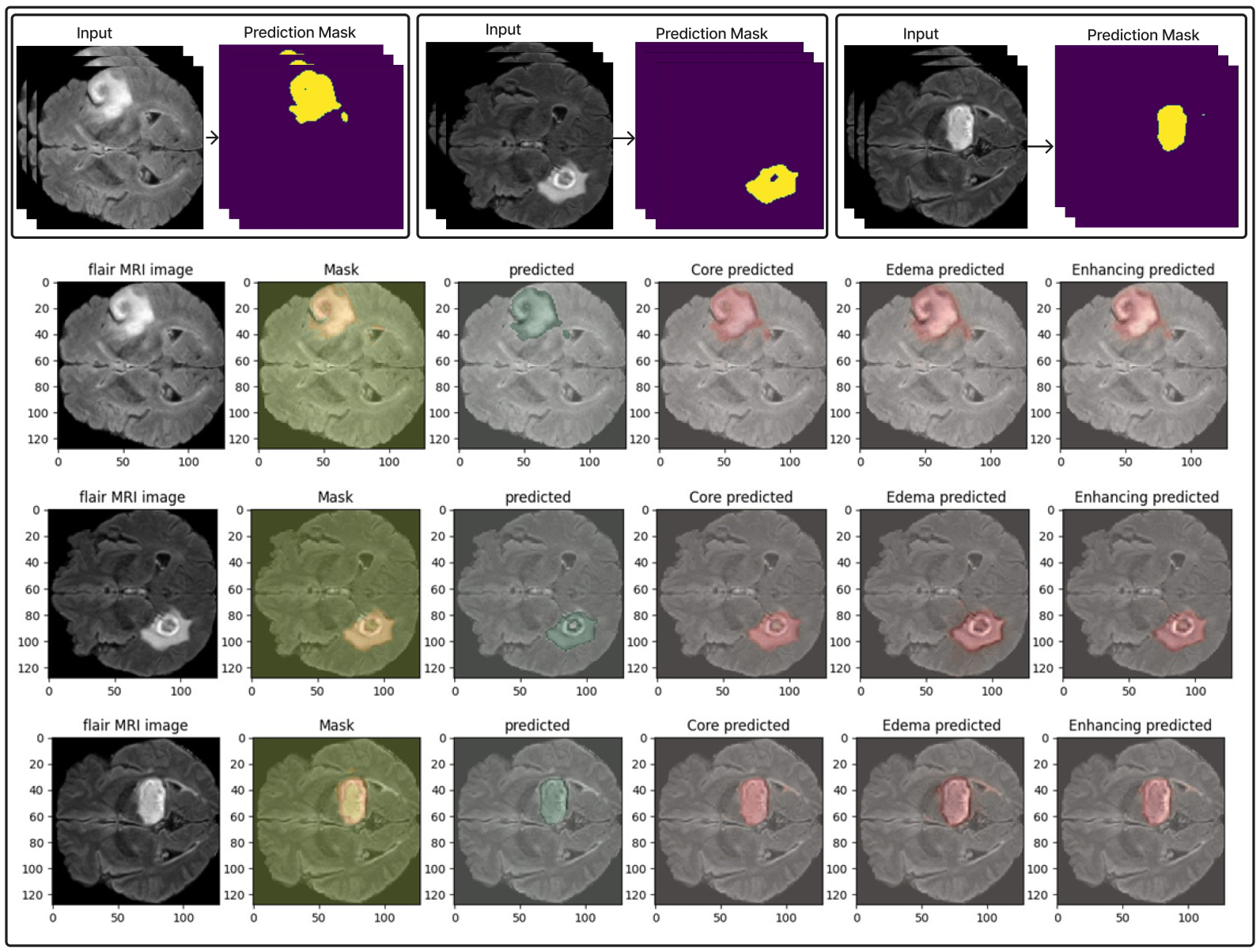

Supplement: Supplementary Figure 1 — Example of segmentation results with just T1 modality input. [file Data_Sheet_1.zip › Supplementary Material/Figure 4.png]

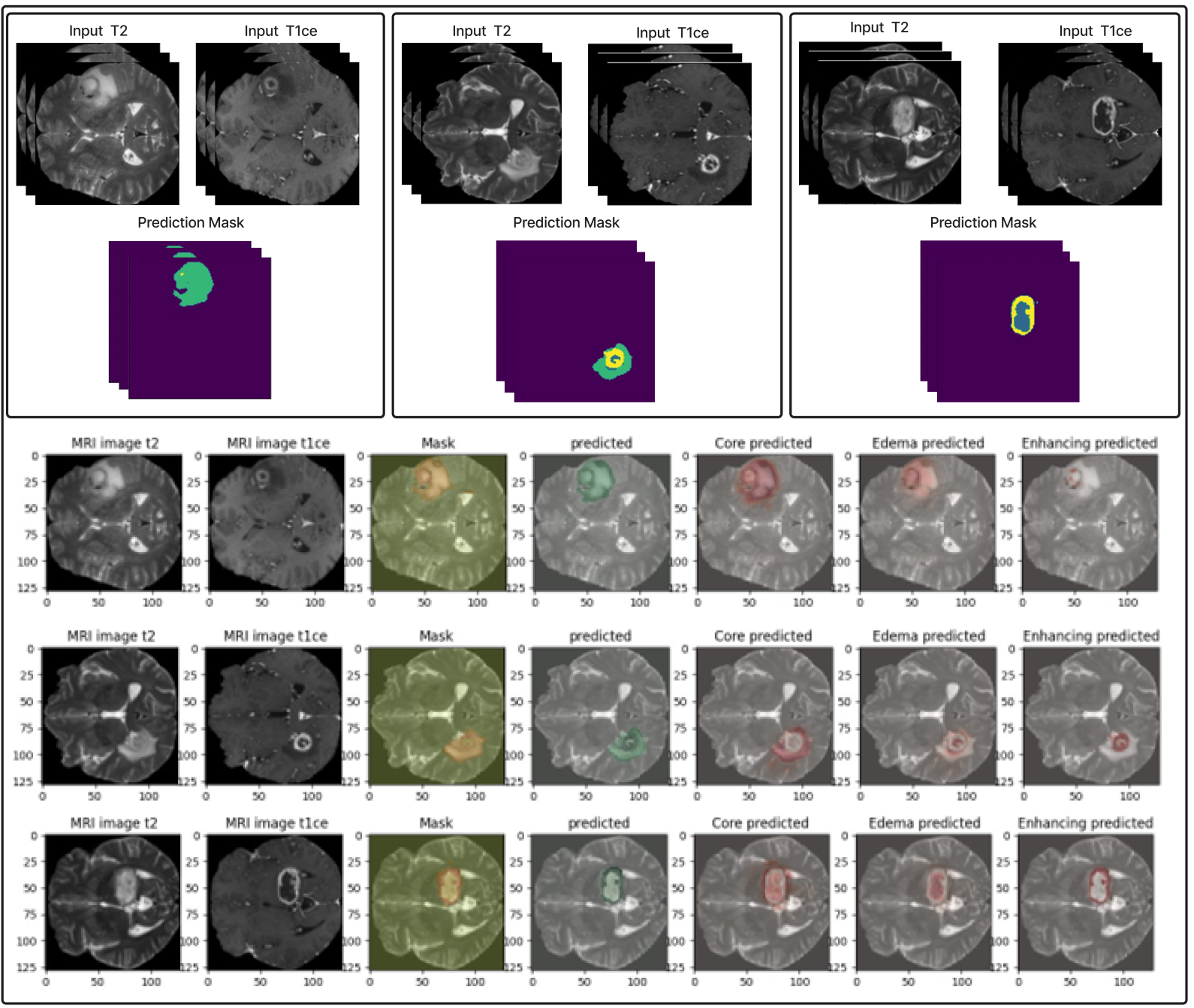

Supplement: Supplementary Figure 1 — Example of segmentation results with just T1 modality input. [file Data_Sheet_1.zip › Supplementary Material/Figure 5.png]

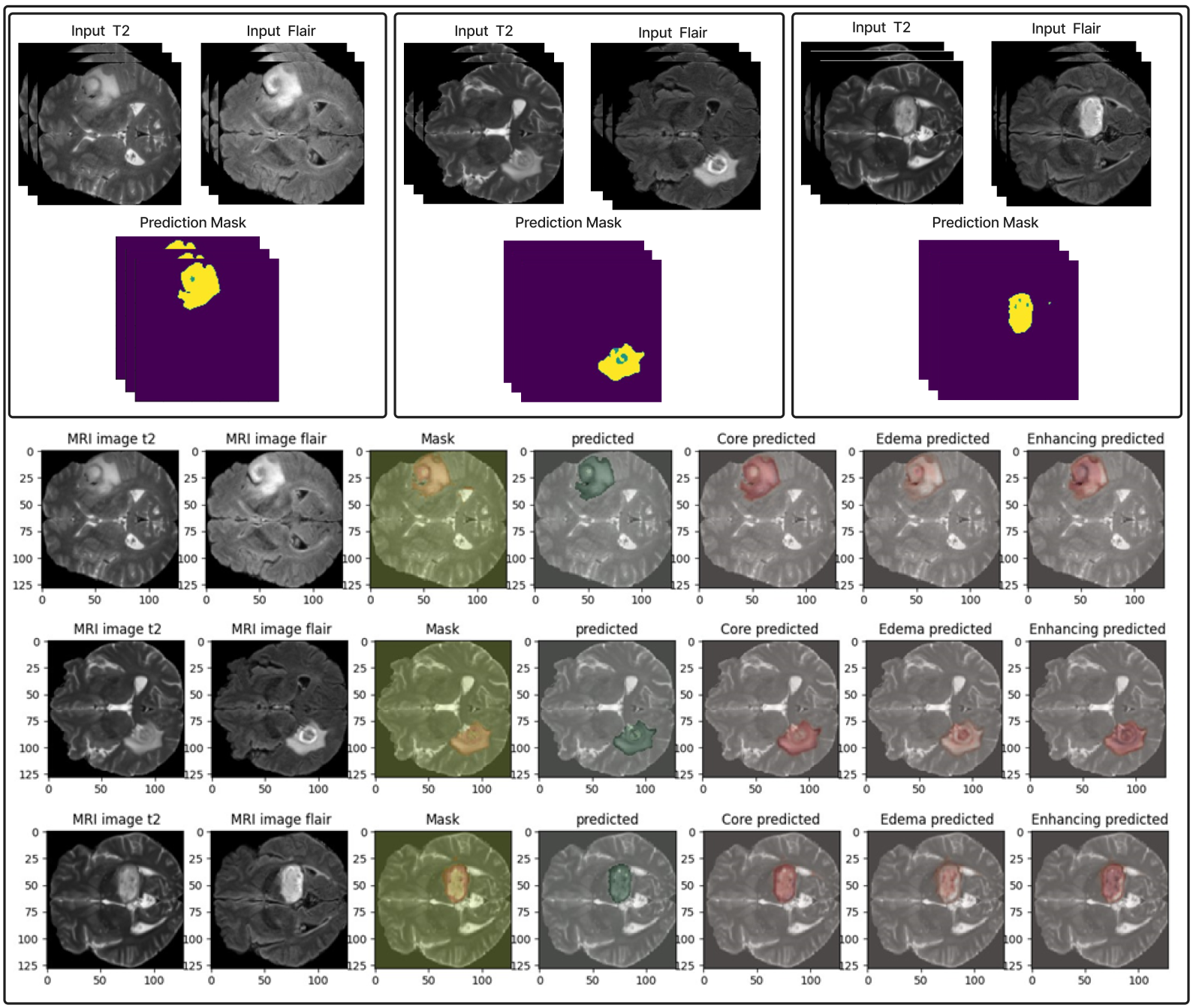

Supplement: Supplementary Figure 1 — Example of segmentation results with just T1 modality input. [file Data_Sheet_1.zip › Supplementary Material/Figure 6.png]

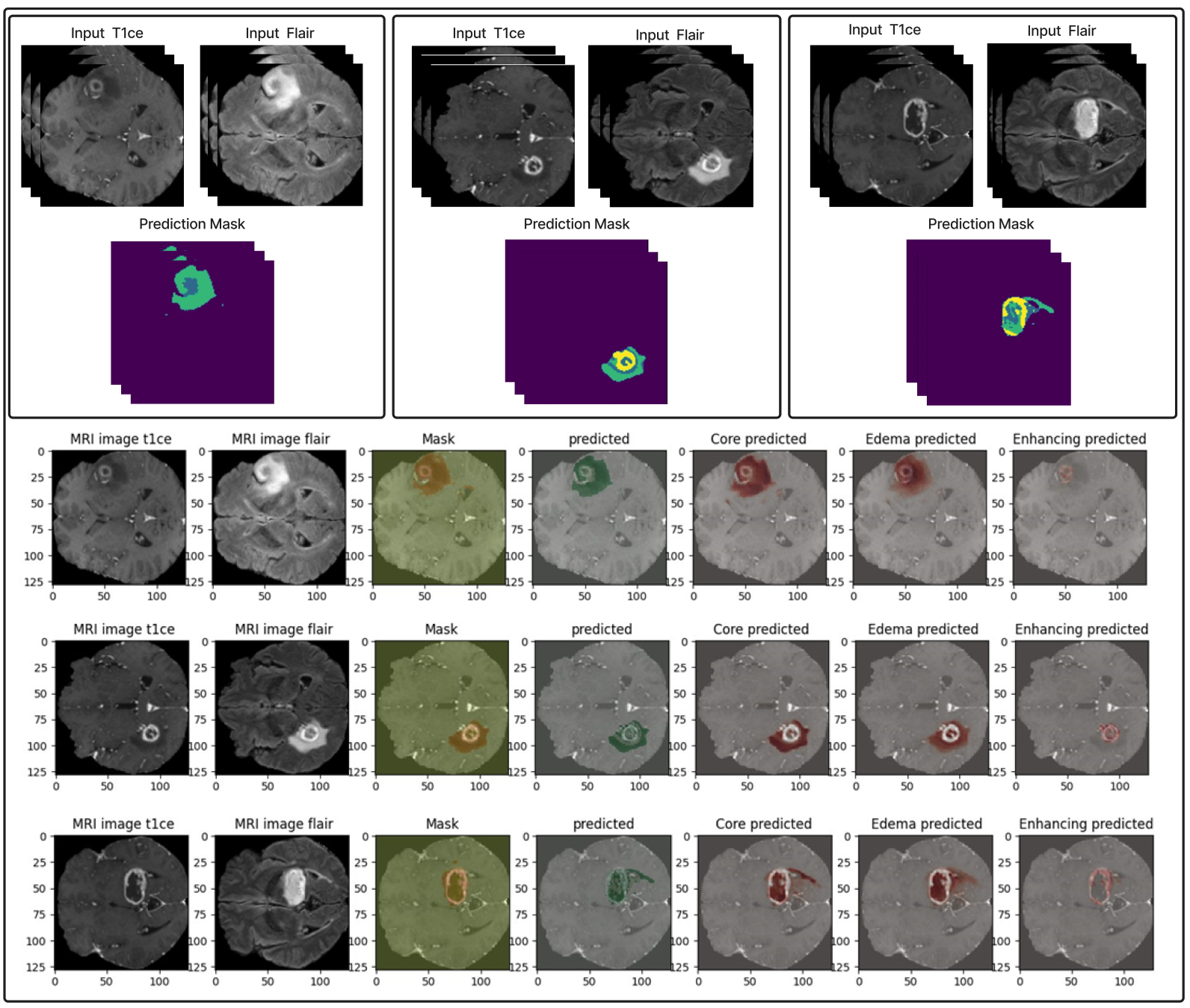

Supplement: Supplementary Figure 1 — Example of segmentation results with just T1 modality input. [file Data_Sheet_1.zip › Supplementary Material/Figure 7.png]

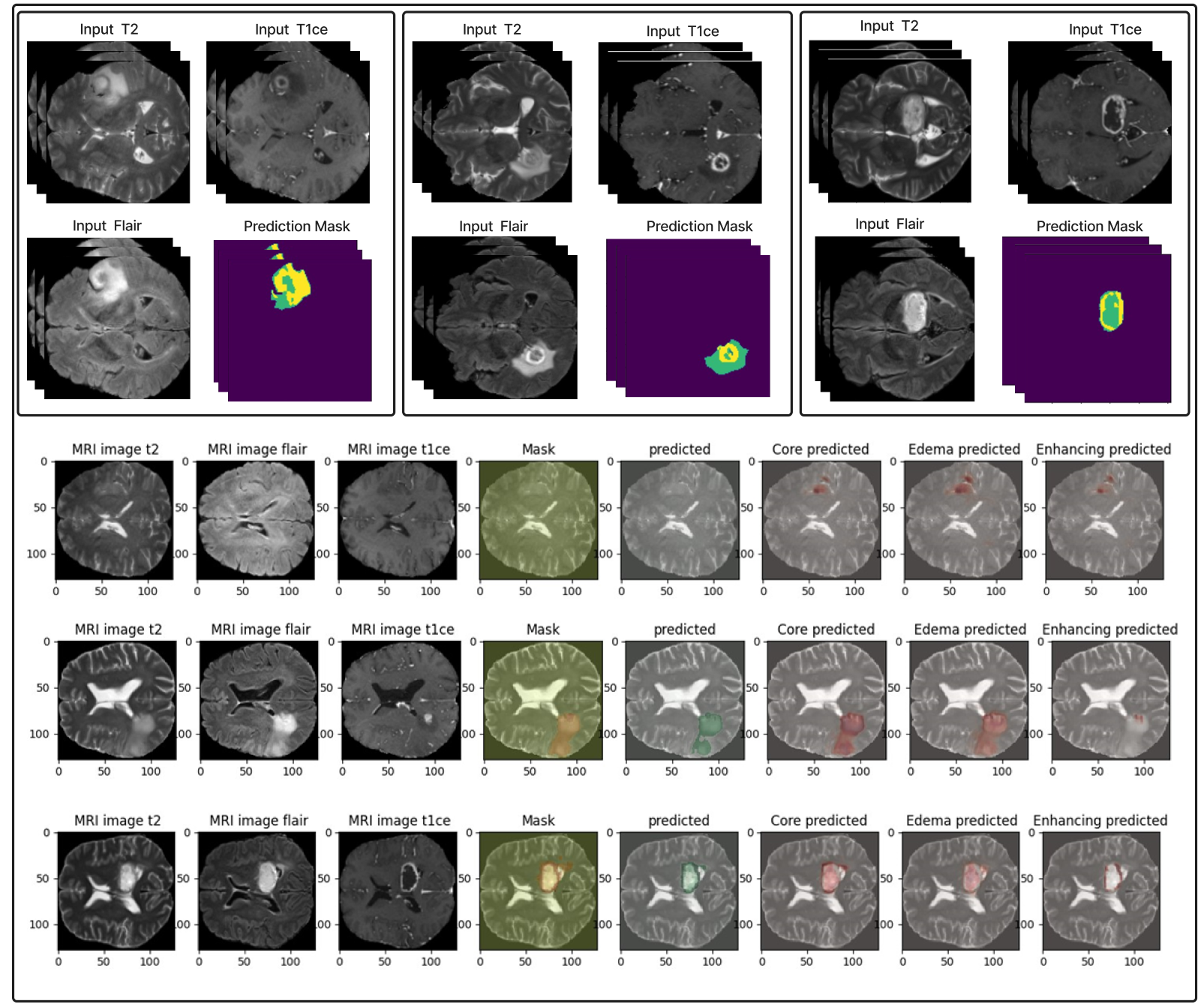

Supplement: Supplementary Figure 1 — Example of segmentation results with just T1 modality input. [file Data_Sheet_1.zip › Supplementary Material/Figure 8.png]

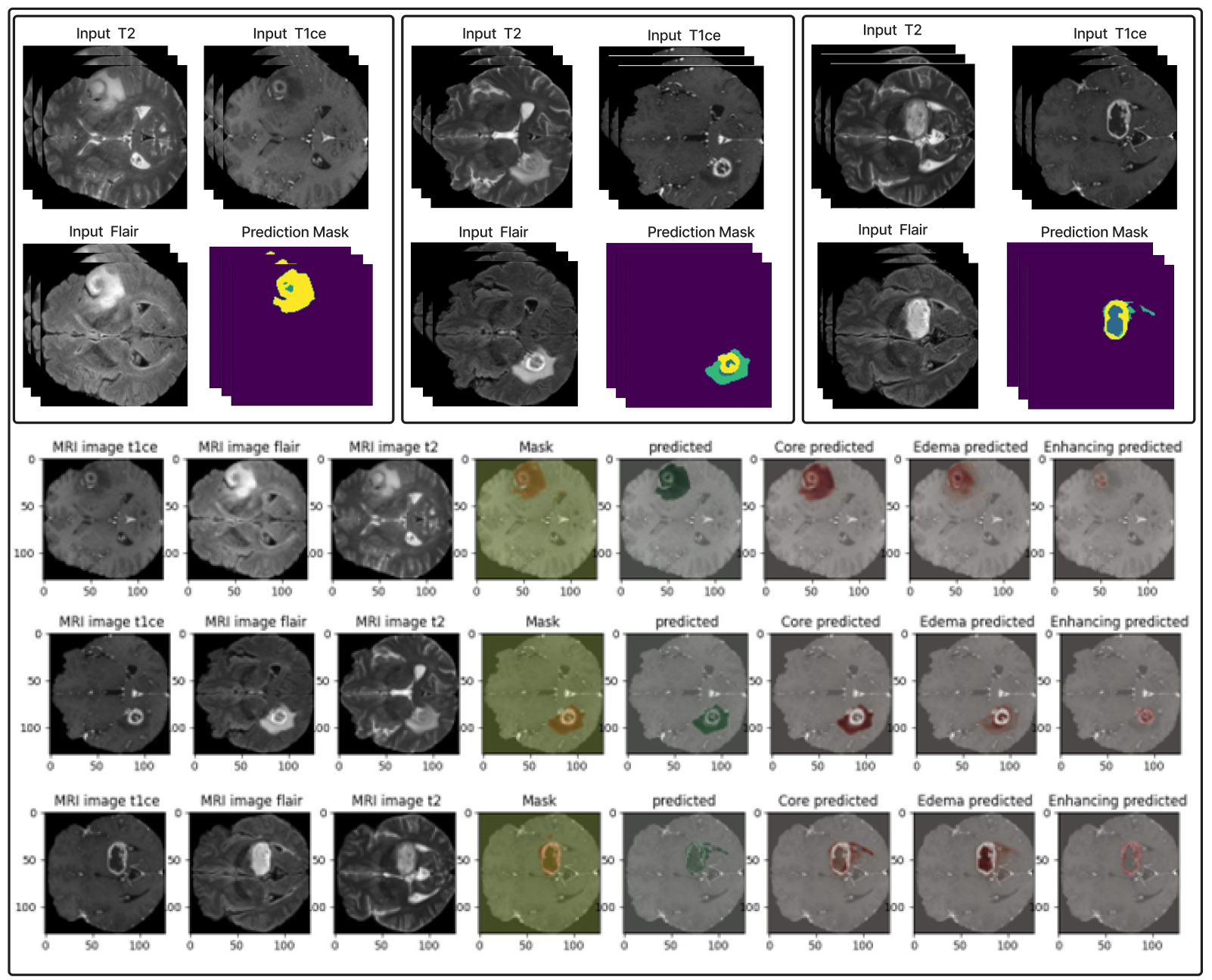

Supplement: Supplementary Figure 1 — Example of segmentation results with just T1 modality input. [file Data_Sheet_1.zip › Supplementary Material/Figure 9.png]
